# Therapy-induced senescent tumor cell-derived extracellular vesicles promote colorectal cancer progression through SERPINE1-mediated NF-κB p65 nuclear translocation

**DOI:** 10.1186/s12943-024-01985-1

**Published:** 2024-04-04

**Authors:** Dan Zhang, Jian-Wei Zhang, Hui Xu, Xin Chen, Yu Gao, Huan-Gang Jiang, You Wang, Han Wu, Lei Yang, Wen-Bo Wang, Jing Dai, Ling Xia, Jin Peng, Fu-Xiang Zhou

**Affiliations:** 1 Department of Radiation and Medical Oncology, Zhongnan Hospital of Wuhan University, Wuhan, Hubei 430071 China; 2 Hubei Key Laboratory of Tumor Biological Behaviors, Zhongnan Hospital, Wuhan University, Wuhan, China; 3 Hubei Clinical Cancer Study Center, Zhongnan Hospital, Wuhan University, Wuhan, China; 4 Department of Gastroenterology, The First Affiliated Hospital of Chongqing Medical University, Chongqing, China

**Keywords:** Cellular senescence, Extracellular vesicles, Colorectal cancer, SERPINE1, p65

## Abstract

**Background:**

Cellular senescence frequently occurs during anti-cancer treatment, and persistent senescent tumor cells (STCs) unfavorably promote tumor progression through paracrine secretion of the senescence-associated secretory phenotype (SASP). Extracellular vesicles (EVs) have recently emerged as a novel component of the SASP and primarily mediate the tumor-promoting effect of the SASP. Of note, the potential effect of EVs released from STCs on tumor progression remains largely unknown.

**Methods:**

We collected tumor tissues from two cohorts of colorectal cancer (CRC) patients to examine the expression of p16, p21, and SERPINE1 before and after anti-cancer treatment. Cohort 1 included 22 patients with locally advanced rectal cancer (LARC) who received neoadjuvant therapy before surgical resection. Cohort 2 included 30 patients with metastatic CRC (mCRC) who received first-line irinotecan-contained treatment. CCK-8, transwell, wound-healing assay, and tumor xenograft experiments were carried out to determine the impacts of EVs released from STCs on CRC progression in vitro and in vivo. Quantitative proteomic analysis was applied to identify protein cargo inside EVs secreted from STCs. Immunoprecipitation and mass spectrometer identification were utilized to explore the binding partners of SERPINE1. The interaction of SERPINE1 with p65 was verified by co-immunoprecipitation, and their co-localization was confirmed by immunofluorescence.

**Results:**

Chemotherapeutic agents and irradiation could potently induce senescence in CRC cells in vitro and in human CRC tissues. The more significant elevation of p16 and p21 expression in patients after anti-cancer treatment displayed shorter disease-free survival (DFS) for LARC or progression-free survival (PFS) for mCRC. We observed that compared to non-STCs, STCs released an increased number of EVs enriched in SERPINE1, which further promoted the progression of recipient cancer cells. Targeting SERPINE1 with a specific inhibitor, tiplaxtinin, markedly attenuated the tumor-promoting effect of STCs-derived EVs. Additionally, the patients with greater increment of SERPINE1 expression after anti-cancer treatment had shorter DFS for LARC or PFS for mCRC. Mechanistically, SERPINE1 bound to p65, promoting its nuclear translocation and subsequently activating the NF-κB signaling pathway.

**Conclusions:**

We provide the in vivo evidence of the clinical prognostic implications of therapy-induced senescence. Our results revealed that STCs were responsible for CRC progression by producing large amounts of EVs enriched in SERPINE1. These findings further confirm the crucial role of therapy-induced senescence in tumor progression and offer a potential therapeutic strategy for CRC treatment.

**Supplementary Information:**

The online version contains supplementary material available at 10.1186/s12943-024-01985-1.

## Introduction

Colorectal cancer (CRC) is one of the most morbid and deadly malignancies worldwide [1]. Chemotherapy, radiotherapy, and targeted therapy have been widely applied in the clinical management of patients with CRC. However, the therapeutic effects are unsatisfactory as patients frequently experience tumor relapse and metastasis. Hence, understanding the mechanisms underlying this process is crucial for the development of new therapeutic strategies.

Cellular senescence is a state of stable cell cycle arrest that is triggered by various internal or external stimuli, including telomere shortening, oncogenic and genotoxic stress, hypoxia, and nutrient deprivation [2, 3]. Interestingly, cancer cells can be rendered senescent when exposed to anti-cancer treatment such as chemotherapy and radiotherapy, which is termed therapy-induced senescence (TIS) [4, 5]. Although multiple researches in recent years have investigated the association of TIS with tumor progression, in vivo evidence of the clinical prognostic implications of TIS is still lacking [6–8]. It has been shown that senescent tumor cells (STCs) could gradually accumulate in the tumor following anti-cancer treatment. More importantly, STCs remain metabolically active and secrete numerous pro-inflammatory cytokines and chemokines, denominated the senescence-associated secretory phenotype (SASP) [9]. Growing evidence indicates that the SASP secreted by STCs influence the malignant biological behaviors of neighboring cells through paracrine signaling, thus leading to detrimental outcomes [10, 11]. Notably, most of the current research concentrates primarily on cytokines and chemokines of the SASP, little is known about extracellular vesicles (EVs) as a novel component of the SASP.

EVs are small cell-derived membrane-bound vesicles that contain heterogeneous contents, including genetic material, proteins, and lipids [12]. The emerging role of EVs as important mediators in intercellular communication is becoming appreciated, although EVs are initially considered only as a mechanism for eliminating unwanted components from cells. EVs have been reported to be associated with various diseases, including immune responses, neurodegenerative diseases, cardiovascular diseases, and cancer [12–14]. In particular, EVs secreted from cancer cells and cancer-associated stromal cells have been demonstrated to facilitate tumor progression [15]. Intriguingly, several recent studies revealed that in addition to secretory proteins, senescent cells exhibited an increased secretion of EVs [16, 17]. Furthermore, EVs released from senescent cells exert a growth-promoting effect on neighboring cancer cells via enrichment of EphA2 [18]. In addition, senescent stromal cells facilitate chemoresistance through SIRT1 loss-mediated EVs over-production [19]. Of note, most current literature concentrates on the effects of EVs generated from senescent stromal cells. To the best of our knowledge, the cargo and function of STCs-derived EVs in CRC remains unexplored.

In this study, we set out to evaluate the clinical prognostic significance of TIS and the impact of EVs derived from therapy-induced STCs on tumor progression in CRC. We observed that chemotherapeutic agents and irradiation could trigger senescence in CRC cells both in vitro and in vivo. Compared to non-STCs, STCs released a significantly greater number of EVs. In addition, STCs-released EVs promoted tumor progression in vitro and in vivo. Mechanistically, we found that serpin family E member 1 (SERPINE1) was enriched in EVs released by STCs. Through the packaging of EVs, SERPINE1 was transported into recipient cancer cells, bound to NF-κB p65, promoting its nuclear translocation and thus contributing to tumor progression. Targeting SERPINE1 with a specific inhibitor, tiplaxtinin (TPX), markedly attenuated the tumor-promoting effect of STCs-derived EVs. Patients with more significant elevation of p16, p21, and SERPINE1 expression in CRC tissues after anti-cancer treatment displayed shorter disease-free survival (DFS) or progression-free survival (PFS). Therefore, targeting SERPINE1 may be a potential strategy for the treatment of CRC.

## Materials and methods

### Patients and sample collection

This study was approved by the Medical Ethics Committee of Zhongnan Hospital of Wuhan University (Ethics No: 2021004K). To determine whether anti-cancer treatment could induce cancer cell senescence in vivo and evaluate the clinical prognostic significance of TIS, two cohorts of CRC patients at Zhongnan Hospital of Wuhan University from January 2017 to December 2022 were collected.

In cohort 1, we collected paired colonoscopy biopsy specimens and radical resection specimens from 22 patients with locally advanced rectal cancer (LARC). Inclusion criteria: (1) The age range was from 18 to 75 years old, and gender was not restricted; (2) Colonoscopy diagnosed as CRC and the colonoscopic biopsy tissue specimens were available; (3) Neoadjuvant therapy was performed before radical surgery; (4) The radical resection specimens were available. Exclusion criteria: (1) History of radiotherapy and chemotherapy; (2) History of other malignant diseases; (3) History of bowel surgery.

Cohort 2 enrolled 30 patients with metastatic CRC (mCRC). Inclusion criteria: (1) The age range was from 18 to 75 years old, and gender was not restricted; (2) Patients with mCRC received first-line regimen contained irinotecan before surgery or biopsy; (3) The surgical resection specimens or colonoscopy biopsy specimens before anti-cancer treatment were available; (4) The biopsy spacimens or surgically resected tumors after irinotecan-based chemotherapy were available. Exclusion criteria: (1) History of radiotherapy and chemotherapy; (2) History of other malignant diseases; (3) History of bowel surgery.

### Cell culture and senescence induction

Human CRC cell lines HCT116 and RKO, and human embryonic kidney cell line 293T were purchased from Procell Life Science & Technology Company (Wuhan, China). Cells were cultured in DMEM (Gibco, Carlsbad, CA) supplemented with 10% FBS (Gibco, Carlsbad, CA) at 37 °C with 5% CO_2_. For chemotherapy-induced senescence, 1.5 µM irinotecan (CPT-11) in HCT116 cells and 2.5 µM CPT-11 in RKO cells were used. For ionizing radiation (IR)-induced senescence, HCT116 cells were irradiated at different doses using the X-Ray Biological Irradiator (Precision X-Ray, Madison, USA). Cells were cultured for 96 h to allow the development of the senescent phenotype. Untreated CRC cells were cultured for 48 h and served as control.

### Reagents and plasmids

CPT-11 (MCE, Shanghai, China) and TPX (MCE, Shanghai, China) were dissolved in DMSO (Beyotime, Shanghai, China) at the concentration of 40 mM and 50 mM, respectively. The stock solutions of CPT-11 and TPX were stored at -80 °C. Before use, the stock solution of CPT-11 was diluted with DMEM to 1.5 µM (for HCT116 cells) or 2.5 µM (for RKO cells), and the stock solution of TPX was diluted with DMEM to 20 µM. Plasmids of GFP-SERPINE1, p65-FLAG and mock vectors were purchased from Sino Biological (Sino Biological, Beijing, China).

### Senescence-associated β-galactosidase (SA-β-Gal) staining

The activity of SA-β-Gal was determined using SA-β-Gal staining kit (Beyotime, Shanghai, China) according to the manufacturer’s instructions. The cultured cells were gently washed twice with PBS. Cells were fixed with the fixative solution and incubated with the SA-β-Gal staining solution mix overnight at 37 °C in an incubator without CO_2_. The SA-β-Gal staining solution mix was composed of X-Gal, staining solution and staining supplement. Under the catalysis of β-Gal, the X-Gal was generated into dark blue products. After washing with PBS, five random fields were captured under a microscope (Olympus IX73, Tokyo, Japan) for the analysis of the percentage of SA-β-Gal-positive cells.

### CCK-8 assay

Cell viability was detected using CCK-8 assay (Beyotime, Shanghai, China). In brief, 5.0 × 10^3^ CRC cells per well were seeded into 96-well plates. At the indicated time points, 10 µl CCK-8 solution was added into each well and incubated at 37 ℃ for 1 h in the dark. The absorbance was measured at a wavelength of 450 nm by a microplate reader.

### EdU incorporation assay

BeyoClick™ EdU Cell Proliferation Kit with Alexa Fluor 488 (Beyotime, Shanghai, China) was utilized and EdU incorporation assay was performed according to the manufacturer’s protocol. The nuclei were stained with DAPI for 10 min in the dark and images were captured under a fluorescent microscope (Olympus IX73, Tokyo, Japan).

### Colony formation assay

CRC cells were plated in 6-well plates at a density of 2 × 10^5^ cells per well and treated with or without CPT-11 at the indicated concentrations for 96 h. Then, cells were trypsinized and re-seeded into 6-well plates (1.5 × 10^3^ cells per well) and cultured for two weeks. After washing with PBS, cells were fixed with 4% paraformaldehyde (Beyotime, Shanghai, China) for 30 min at room temperature and stained with 0.1% crystal violet (Beyotime, Shanghai, China) for 15 min. The number of cell colonies was counted using Image J software (National Institutes of Health, Bethesda, MD, USA).

### Wound-healing assay

To detect the impact of the EVs on the migration ability of CRC cells, the wound-healing assay was conducted as previously described [20]. Briefly, CRC cells were plated in 6-well plates. When the cells reached 80% confluence, a wound was created by manually scraping the cell monolayer with a 10 µl pipette tip. The cells were washed once with PBS to remove the debris and the medium was replaced with fresh medium (containing 0.5% FBS) supplemented with or without EVs (15 µg/ml). The area of migration was photographed at indicated time points and analyzed using Image J software.

### Transwell assay

The transwell assay was conducted to evaluate the impact of EVs on the migration and invasion of CRC cells. For the migration assay, 5 × 10^4^ CRC cells were seeded into the upper chambers (8 μm pore size, Corning, MA, USA) in serum-free DMEM. The lower chamber was filled with DMEM (2.5% FBS) with or without EVs (15 µg/ml). After incubation at 37 °C for 48 h, the migrated cells were fixed, stained, and photographed under a microscope (Olympus IX73, Tokyo, Japan). For the invasion assay, the upper compartment of the chamber was precoated with Matrigel (Corning, MA, USA) and the other steps were the same as the migration assay.

### Flow cytometry

Cell cycle distribution was determined by flow cytometry as previously described [21]. In Brief, cells were incubated with DNA staining solution and permeabilization solution at room temperature for 30 min in the dark. Then, DNA content was measured by flow cytometry using a FACS Calibur system (Becton Dickinson, Franklin Lakes, NJ, USA), and the data were analyzed with FlowJo FACS analysis software (TreeStar, Ashland, OR, USA).

### RT-qPCR

Total RNA was extracted from cells using Trizol reagent (Takara, Ohtsu, Japan) and reverse transcribed using the reverse transcription kit (Takara, Ohtsu, Japan). RT-qPCR was carried out with the SYBR Green Master Mix (Vazyme, Nanjing, China) on a CFX96 Touch System (Bio-Rad, CA, USA). β-actin was used as an internal control. Relative gene expression was calculated using the 2^−ΔΔCT^ method. The primers were listed in Table S1.

### Western blot

Protein was isolated from harvested cells or EVs by RIPA lysis buffer (Beyotime, Shanghai, China) containing protease inhibitor Cocktail (MCE, Shanghai, China). The protein concentration was determined using the BCA protein assay kit (Beyotime, Shanghai, China) in accordance with the manufacturer’s instructions. Protein extracts were separated in 10% SDS-PAGE and transferred to PDVF membranes (Millipore, MA, USA). After blocking with 5% nonfat milk at room temperature for 2 h, the membranes were washed with TBST and incubated sequentially with primary antibody and secondary antibody. The protein signals were detected by enhanced chemiluminescence with ECL detection reagents on the chemiluminescence imager (Tanon, Shanghai, China). The primary antibodies used were listed in Table S2.

### Immunofluorescent staining

Cells were fixed with 4% paraformaldehyde for 30 min and penetrated with Triton X-100 for 15 min. 10% goat serum (BOSTER, Wuhan, China) was utilized to block at 37 ℃ for 1 h. The cells were incubated sequentially with primary antibody at 4 °C for 16 h and secondary antibody at 37 ℃ for 1 h in the dark. Nuclei were counterstained with DAPI for 10 min. Images were captured by a confocal laser scanning microscope (SP8, Leica, Germany). The primary antibodies used were listed in Table S2.

### Isolation of EVs

For chemotherapy-induced senescence, CRC cells were plated in 100 mm dishes and treated with CPT-11 (1.5 µM CPT-11 in HCT116 cells and 2.5 µM CPT-11 in RKO cells) for 96 h to induce senescence. To inhibit the expression of SERPINE1 in EVs from STCs, CRC cells were simultaneously treated with CPT-11 and TPX (20 µM) for 96 h. For IR-induced senescence, HCT116 cells were plated in 100 mm dishes and exposed to 10 Gy irradiation. Cells were cultured for 96 h to allow the development of the senescent phenotype. For control, HCT116 cells and RKO cells were plated in 10 mm dishes without treatment and cultured for 48 h to reach 90% confluence.

The cultured cells were washed twice with PBS to remove the drug and FBS and cultured in serum-free DMEM for 48 h, then the culture media was collected for EVs isolation and the number of cells in dishes was counted. The EVs were isolated by differential ultracentrifugation as described previously [22, 23]. Briefly, the harvested culture media was centrifuged at 300 *g* for 10 min followed by centrifugation at 2,000 *g* for 20 min to remove cellular debris and apoptotic bodies. The supernatant was transferred and centrifuged at 10,000 *g* for 30 min at 4 ℃ to isolate large EVs. Then the supernatant was filtered through a 0.22 μm polyethersulfone filter (Millipore, Merck Millipore, USA) to reduce potential large EVs contamination. The supernatant filtrate was subjected to high-speed ultracentrifugation at 100,000 *g* for 70 min at 4 ℃ with an SW28 rotor (Optima XE-100, Beckman Coulter, USA). Then the pellets were washed twice with PBS and ultracentrifuged at 100,000 *g* for 70 min at 4 ℃. The purified EVs were resuspended in sterile PBS and used for the following experiments.

### Nanoparticle tracking analysis (NTA)

The concentration and size distribution of EVs were measured using a ZetaView nanoparticle tracking analyser (Particle Metrix, Meerbusch, Germany) equipped with ZetaView software (version 8.05.14 SP7). EVs were diluted to the appropriate concentration in PBS prior to measurement. The ZetaView system was calibrated using 110 nm polystyrene particles. NTA measurement was recorded and analyzed at 11 positions. The average concentration from three recordings was used as the EV concentration.

### Transmission Electron microscopy (TEM)

The EVs samples were diluted in PBS before measurement. The EVs samples were added dropwise on 200 square mesh TEM grids and incubated for 10 min at 37 ℃. The grids were negatively stained with 2% phosphotungstic acid for 3 min, and imaged on a JEM1400 transmission electron microscope.

### Proteinase K assay

EVs isolated from senescent HCT116 cells were resuspended in PBS and split into three identical aliquots. The mix was incubated at 37 ℃ with 0.5 mg/ml proteinase K in the presence or absence of 1% Triton X-100 for 30 min. For control, one of the aliquots was incubated without proteinase K. The samples were then boiled for 10 min and analyzed by western blot.

### PKH67-labeled EVs transfer assay

CRC cells (5.0 × 10^4^) were plated into 24-well plate. EVs (10 µg) released from senescent CRC cells were labeled with a PKH67 green fluorescence labeling kit (Sigma-Aldrich) [24]. The excess dye was removed through washing with PBS. The EVs were collected again by ultracentrifugation and resuspended in PBS. CRC cells were incubated with labeled EVs. After 18 h, cells were washed twice with PBS and stained with Hoechst 33342 for 10 min in the dark. Then cells were photographed under a fluorescent microscope (Olympus BX53, Tokyo, Japan).

### iTRAQ-labeling quantitative proteomics analysis

EVs samples were isolated from senescent HCT116 cells and non-senescent HCT116 cells. Lysis buffer (7 M urea, 2 M thiourea, 4% CHAPS, 40 mM Tris-HCl, pH 8.5) with 1 × Cocktail (with EDTA) was added into each EVs sample. The mixtures were reduced with 10 mM DTT (37℃ for 30 min) and alkylated with 55 mM iodoacetamide (45 min in the dark). The samples were precipitated with ice-cold acetone (1:5, v/v) at -20 ℃ for 2 h and centrifuged at 25,000 *g* for 15 min. The pellets were air-dried and resuspended in lysis buffer followed sonicating (frequency of 60 Hz, for 2 min). The samples were centrifuged again, and the supernatant was transferred to a new tube. Protein quantitation was performed using a Bradford Protein Assay Kit.

The iTRAQ-based proteomic analysis was conducted by Beijing Genomics Institute. Briefly, proteins (100 µg) of each sample were digested using trypsin (1:20 w/w, Promega, Madison, USA) at 37 ℃ for 4 h. The digested protein peptide was desalted and labeled with iTRAQ reagents according to the kit protocol (Applied Biosystems, Foster City, USA). Then the peptides were pooled and purified using a 5 μm 4.6 × 250 mm Gemini C18 column with an LC-20AB liquid phase system (Shimadzu, Tokyo, Japan). The dried peptide samples were reconstituted with buffer A (5% ACN pH 9.8) and injected, eluting at a flow rate of 1mL/min by following gradients: 5% buffer B (95% ACN, pH 9.8) for 10 min, 5–35% for 40 min, 35–95% for 1 min, buffer B for 3 min, and 5% buffer B for 10 min. Finally, the eluted peptides were pooled into 20 fractions, which were then freeze-dried. The dried peptide samples were reconstituted with buffer A (2% ACN, 0.1% FA) for LC-MS/MS analysis. Separation was performed by Thermo UltiMate 3000 UHPLC. Peptides were separated with a gradient from buffer B (98% ACN, 0.1% FA) and the liquid gradient setting: 0–5 min, 5% buffer B; 5–45 min, 5–25% buffer B; 45–50 min, 25–35% buffer B; 50–52 min, 35–80% buffer B; 52–54 min, 80% buffer B; 54–60 min, 5% buffer B. Ultimately, the separated peptides were analyzed in Q Exactive HF-X (Thermo Fisher Scientific, San Jose, CA) for DDA (Data Dependent Acquisition) mode detection. Spectra generated by the Orbitrap was optimized by automatic gain control (AGC). The AGC was set to: MS1 3E6, MS2 1E5. The main parameters were set: ion source voltage was set to 1.9 kV, MS1 scanning range was 350–1,500 m/z; resolution was set to 60,000; MS2 starting m/z was fixed at 100; resolution was 15,000.

The raw MS/MS data were converted into MGF format by thermo scientific tool Proteome Discoverer, and the exported MGF files were searched using Mascot version 2.3.02 (Matrix Science, London, UK) against human Uniprot database (http://www.uniprot.org). The mass tolerance for precursor ions was set as 10 ppm and the mass tolerance for fragment ions was set as 0.02 Da. All identified proteins were required to have at least 2 peptides with at least one unique peptide. For quantitatively analyzing the peptides labeled with iTRAQ tags, the IQuant software was utilized. Proteins with fold change > 1.5 and Q value < 0.05 were considered differentially expressed.

### Immunoprecipitation (IP) and mass spectrometer (MS) identification

Cells were lysed in IP lysis buffer, followed by incubation on ice for 15 min. Then the cell lysates were centrifuged at 12,000 *g* for 10 min. The supernatant was collected and incubated at 4 ℃ with primary antibody by a roller shaker overnight. Then 40 µl Protein A/G magnetic beads (MCE, Shanghai, China) were incubated with the cell lysates for 2 h at 4 °C. Beads were washed with PBST (PBS with 0.5% Triton X-100) three times. IP proteins were analyzed by western blot or LC-MS/MS analysis. The primary antibodies used were listed in Table S2.

### Tumor xenograft experiments

The animal experiments were performed according to the approved study protocols by the Animal Ethics Committee of Wuhan University (approval number: ZN2023019). 5-week-old male BALB/c nude mice were housed and fed in specific pathogen free conditions. The mice were randomly assigned to four groups: PBS, Ctrl-EVs, Sen-EVs, and TPX-Sen-EVs. HCT116 cells (1 × 10^6^ cells) together with or without EVs (50 µg for each mouse) were injected subcutaneously under the right armpits of the nude mice. The mice were intratumorally injected with EVs (50 µg for each mouse) every other day when the tumor volume reached 50 mm^3^. Mice injected with an equivalent volume of PBS following the same procedure served as control. Tumor volume calculations were obtained every 2 days using the following formula: Tumor volume (mm^3^) = (width^2^ × length)/2. To minimize animal suffering, once the tumor size of each group reached a significant difference, the mice were euthanized and tumors were excised and weighed. The xenograft tissues were fixed with 4% paraformaldehyde and embedded in paraffin for hematoxylin-eosin (H&E) and immunohistochemistry (IHC) staining.

### H&E and IHC staining

For H&E staining, tumor tissues were fixed in formalin, embedded in paraffin, and cut into 5-µm-thick sections followed by H&E standard staining as described previously [21]. Images were taken under a microscope (Olympus BX53, Tokyo, Japan).

IHC staining was carried out as described previously [21]. Briefly, antigen retrieval was conducted by boiling the tissue sections with citrate buffer. Next, tissue sections were blocked in 10% goat serum for 1 h and incubated sequentially with primary antibody at 4 °C overnight and secondary antibody at 37 ℃ for 30 min. Images were captured on a microscope. The expression levels of p16, p21 and SERPINE1 were evaluated according to the immunoreactive score (IRS). The IRS was determined by the multiplication of staining distribution (0, less than 5%; 1, 5–25%; 2, 26–50%; 3, 51–75%; 4, more than 76%) and intensity score (0, no coloration; 1, pale yellow; 2, yellow; and 3, dark brown). IRS > 2 was considered positive. The primary antibodies used were listed in Table S2.

### Statistical analysis

All statistical analyses were carried out with GraphPad Prism 8.0 (GraphPad Software, CA, USA). Categorical data were presented as numbers and proportions and analyzed with chi-square test. Continuous variables were expressed as mean ± standard deviation from at least three independent experiments. The comparison between two groups was conducted using Student’s *t*-test. One-way ANOVA with Bonferroni correction was used for multiple comparisons. Kaplan-Meier curve and log-rank test were used to calculate survival profiles. *p* value < 0.05 was considered statistically significant. *p < 0.05, **p < 0.01, and ***p < 0.001.

## Results

### Therapy-induced cancer cell senescence was observed in human CRC tissues and CRC cell lines

**Fig. 1:**
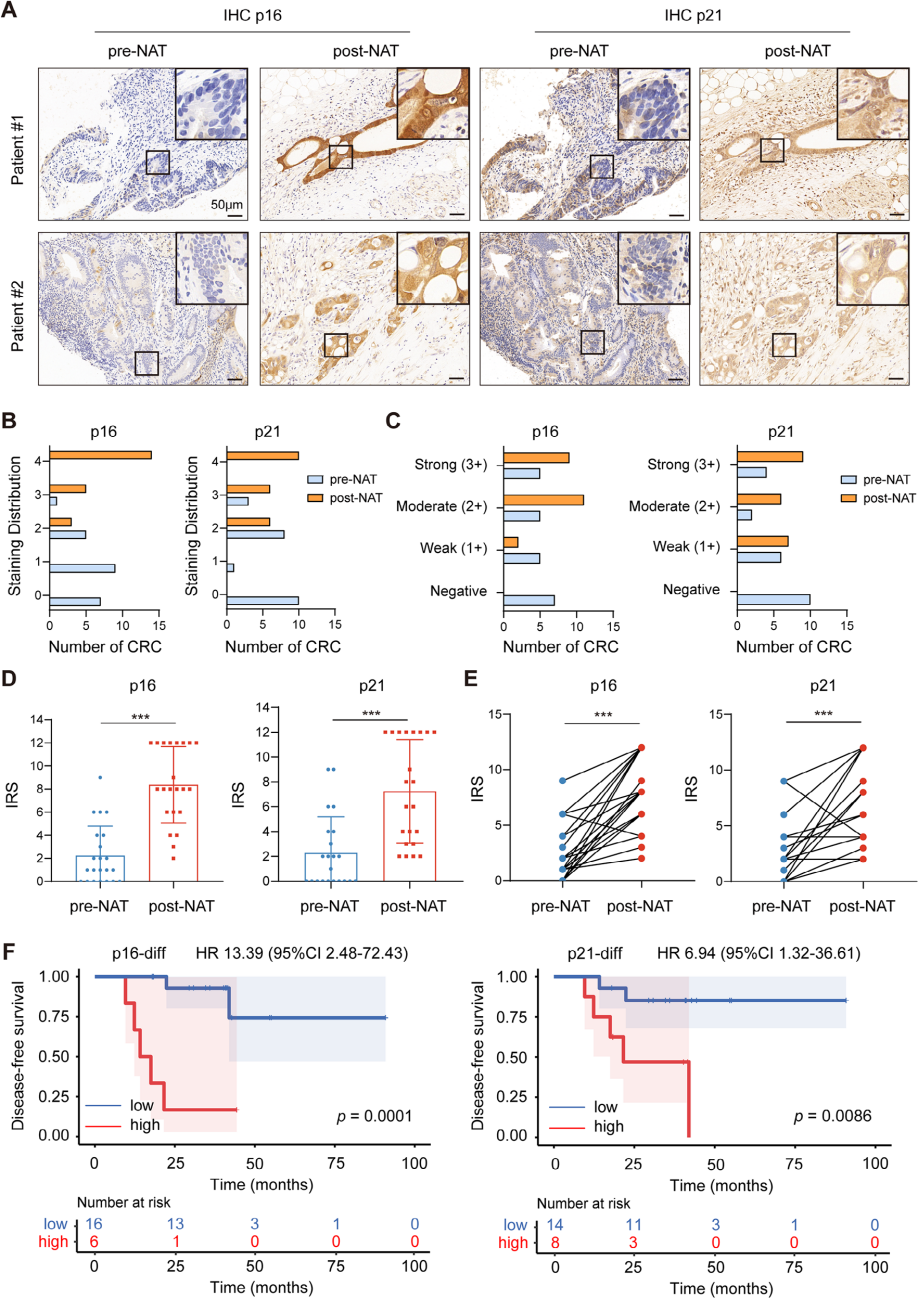
Therapy-induced cancer cell senescence was observed in human LARC tissues of cohort 1. **(A)** IHC staining of p16 and p21 in 22 matched LARC tissues. pre-NAT, colonoscopic biopsy specimens before NAT. post-NAT, surgical specimens after NAT. **(B)** The staining distribution of p16 and p21. **(C)** The intensity score of p16 and p21. **(D** and **E)** The IRS of p16 and p21. **(F)** Kaplan-Meier plots of DFS. The plot was generated according to the difference between pre-NAT IRS and post-NAT IRS of p16 (p16-diff) and p21 (p21-diff). *p < 0.05, **p < 0.01, and ***p < 0.001

To confirm therapy-induced cellular senescence in vivo, 22 patients with LARC who received neoadjuvant therapy (NAT) prior to radical resection were included, and their colonoscopic biopsy specimens before NAT (pre-NAT) and matched surgical specimens after NAT (post-NAT) were collected (cohort 1). The clinicopathologic features of the patients were listed in Table S3. IHC staining demonstrated a significantly greater expression of senescence-specific markers p16 and p21 in post-NAT group compared to the pre-NAT group (Fig. 1A). In comparison with the pre-NAT group, the staining distribution score and intensity of p16 and p21 were noticeably higher in the post-NAT group (Fig. 1B and C). Consistent with this, the IRS analysis showed a dramatic increase of p16 and p21 in the post-NAT group compared to the pre-NAT group (Fig. 1D and E). Additionally, we found that the percentage of p16-positive patients in the post-NAT group was markedly elevated compared to the pre-NAT group, as was the case for p21 (Table 1). The clinical significance of TIS was assessed by calculating the difference between pre-NAT IRS and post-NAT IRS of p16 (p16-diff) and p21 (p21-diff). Kaplan-Meier plots showed that the higher p16-diff and p21-diff correlated with reduced DFS (Fig. 1F).

**Table 1:** p16 and p21 expression in LARC tissues (cohort 1)

Factors	pre-NAT (*n* = 22)	post-NAT (*n* = 22)	χ^2^	*p* value
p16 positive	7 (31.82%)	21 (95.45%)	16.60	<0.001
p16 negative	15 (68.18%)	1 (4.55%)		
p21 positive	7 (31.82%)	18 (81.82%)	9.26	0.002
p21 negative	15 (68.18%)	4 (18.18%)		

Data were analyzed by χ^2^ test with Yate’s correction

**Fig. 2:**
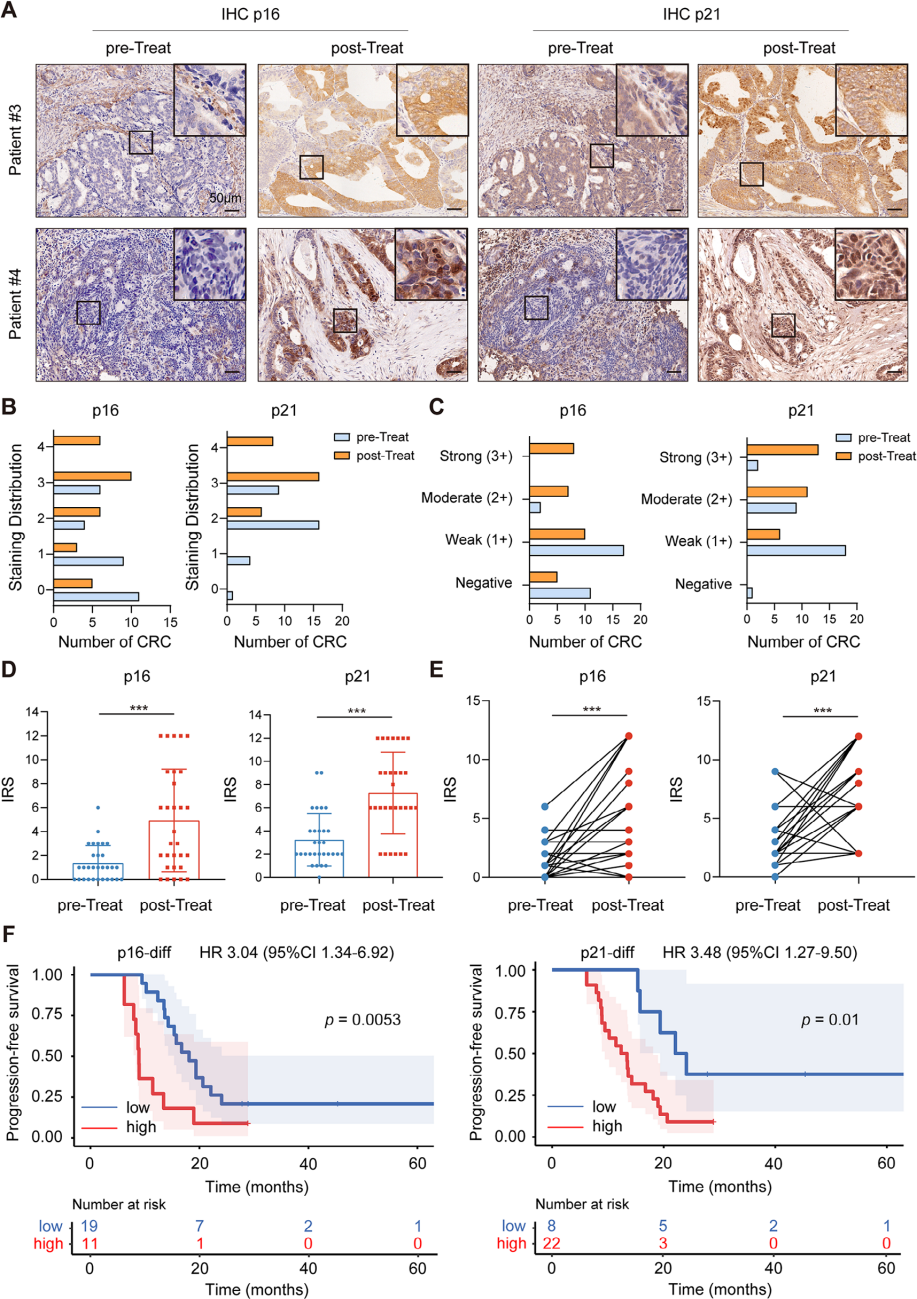
The expression of p16 and p21 was significantly elevated after irinotecan-based chemotherapy in mCRC patients of cohort 2. **(A)** IHC staining of p16 and p21 in matched CRC tissues. pre-Treat, colonoscopy biopsies or surgically resected tumors before anti-cancer treatment. post-Treat, surgically resected tumors after irinotecan-based chemotherapy. **(B)** The staining distribution of p16 and p21. **(C)** The intensity score of p16 and p21. **(D** and **E)** The IRS of p16 and p21. **(F)** Kaplan-Meier plots of PFS. The plot was generated according to the difference between pre-Treat IRS and post-Treat IRS of p16 (p16-diff) and p21 (p21-diff). *p < 0.05, **p < 0.01, and ***p < 0.001

To further confirm the clinical significance of TIS, we collected 30 patients with mCRC received first-line regimen contained irinotecan (cohort 2). The colonoscopy biopsies or surgically resected tumors before anti-cancer treatment were defined as the pre-Treat group. The surgically resected tumors after irinotecan-based chemotherapy were defined as the post-Treat group. The clinicopathologic features of the patients were listed in Table S4. The staining distribution score and intensity of p16 and p21 were noticeably higher in the post-Treat group than in the pre-Treat group (Fig. 2A-C). The IRS of p16 and p21 in the post-Treat group significantly increased compared to the pre-Treat group (Fig. 2D and E). Furthermore, the percentage of p16-positive patients in the post-Treat group was markedly elevated compared to the pre-Treat group, as was the case for p21 (Table 2). The difference between pre-Treat IRS and post-Treat IRS of p16 (p16-diff) and p21 (p21-diff) was calculated. Kaplan-Meier plots revealed that the p16-diff and p21-diff negatively correlated with PFS (Fig. 2F). Altogether, these results indicated that anti-cancer treatment could induce senescence in CRC cells in vivo, and TIS was associated with poor prognosis.

**Table 2:** p16 and p21 expression in mCRC tissues (cohort 2)

Factors	pre-Treat (*n* = 30)	post-Treat (*n* = 30)	χ^2^	*p* value
p16 positive	7 (23.33%)	17 (56.67%)	6.94	0.008
p16 negative	23 (76.67%)	13 (43.33%)		
p21 positive	16 (53.33%)	24 (80.00%)	4.80	0.029
p21 negative	14 (46.67%)	6 (20.00%)		

Data were analyzed by Pearson’s χ^2^ test

**Fig. 3:**
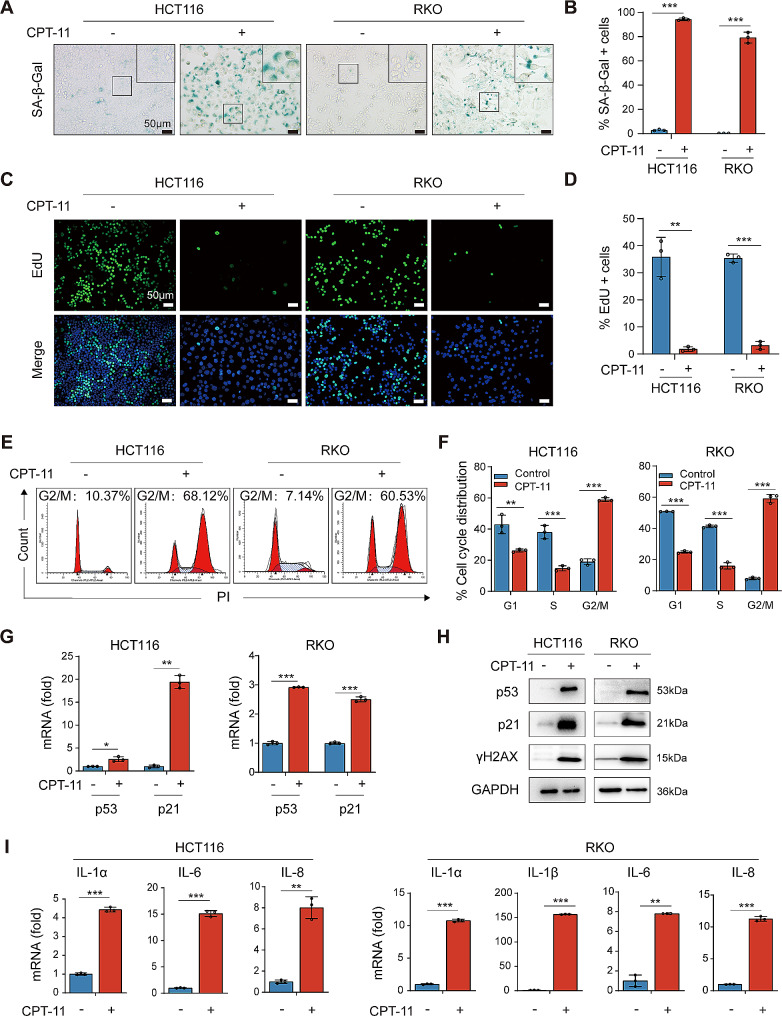
CPT-11 induced senescence in CRC cells. CRC cells were treated with or without CPT-11 for 96 h. **(A)** Representative photographs of SA-β-Gal staining. **(B)** Quantification of SA-β-Gal-positive cells. **(C)** Representative photographs of EdU staining. Green fluorescence indicated EdU staining, and blue fluorescence reflected nuclear staining with DAPI. **(D)** Quantification of EdU-positive cells. **(E)** Flow cytometry analysis of cell cycle distribution. **(F)** Quantification of cell cycle distribution. **(G)** p53 and p21 mRNA levels. **(H)** p53, p21, and γH2AX protein levels. **(I)** mRNA levels of SASP factors. Data represented the mean ± standard deviation of at least 3 independent experiments. *p < 0.05, **p < 0.01, and ***p < 0.001

To assess whether chemotherapeutic agents could trigger senescence in CRC cells in vitro, CPT-11, an inhibitor of DNA topoisomerase I that has been commonly applied in CRC patients, was utilized to treat p53-wild type CRC cell lines HCT116 and RKO. CCK-8 assay showed that CPT-11 significantly decreased cell viability of CRC cells in a concentration-dependent manner (Fig. S1A). We observed that after 96 h of treatment with CPT-11 (HCT116 cells treated with 1.5 µM CPT-11 and RKO with 2.5 µM CPT-11), about 80% of CRC cells exhibited an enlarged and flattened morphological alteration, which was a typical feature of cellular senescence [25]. To further determine the induction of senescence, we evaluated the activity of SA-β-Gal, a widely recognized marker of cellular senescence. CPT-11 significantly increased the proportion of SA-β-Gal-positive cells (Fig. 3A and B). Notably, this concentration of CPT-11 significantly inhibited the growth of CRC cells, while failed to activate cellular apoptosis as evidenced by the unchanged expression of cleaved PARP and cleaved Caspase-3 between the CPT-11-treated group and the control group (Fig. S1B and C). Therefore, in the following experiments in vitro, 1.5 µM CPT-11 in HCT116 cells and 2.5 µM CPT-11 in RKO cells were used to induce senescence unless otherwise indicated.

To further validate the induction of senescence, we next performed EdU incorporation assay and colony formation assay to monitor cell proliferation. The proportion of EdU-positive cells was remarkably reduced after treatment with CPT-11 (Fig. 3C and D). Additionally, a substantial reduction of colony formation was observed in CRC cells treated with CPT-11 (Fig. S1D and E). These results indicated an impaired proliferation activity in CPT-11-treated CRC cells. Cell cycle arrest is another important characteristic of senescent cells. Therefore, we investigated the cell cycle distribution of CPT-11-treated CRC cells. The result of flow cytometry analysis revealed that almost 60% of CPT-11-treated cells were arrested at the G2/M phase (Fig. 3E and F). Since DNA damage response is crucial in the induction of cellular senescence, we evaluated the DNA damage marker γH2AX and senescence-associated p53/p21 pathway. The elevated expression of p53, p21, and γH2AX was observed in CPT-11-treated CRC cells (Fig. 3G and H). Furthermore, the SASP is regarded as one of the most crucial characteristics of senescent cells. Therefore, we investigated the expression of SASP factors and found that CPT-11 treatment led to a significant increase in the mRNA levels of IL-1α, IL-1β, IL-6, and IL-8 (of note, IL-1β was undetectable in HCT116 cells) (Fig. 3I). Additionally, we evaluated the induction of senescence in CRC cells after exposure to IR. IR-treated HCT116 cells exhibited increased SA-β-Gal positivity and elevated expression of p53, p21, and SASP factors, which were consistent with CPT-11-treated CRC cells (Fig. S2A-D). Above all, these results suggested that chemotherapeutic agents and IR could induce senescence in CRC cells in vitro.

### STCs released a significantly increased number of EVs

**Fig. 4:**
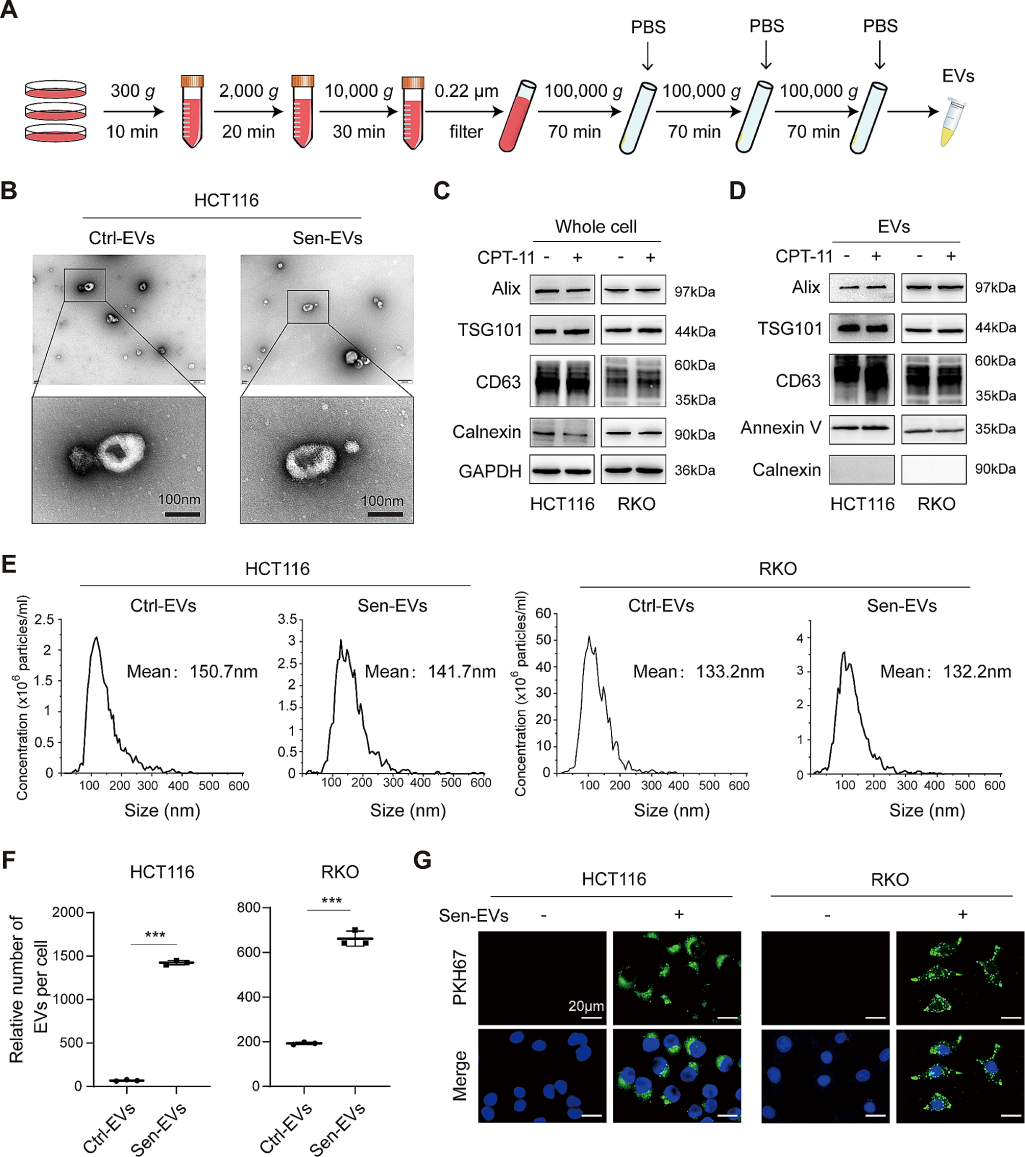
STCs released a significantly increased number of EVs. **(A)** The overview scheme of the EVs isolation. **(B)** Representative TEM images of Ctrl-EVs and Sen-EVs. Ctrl-EVs, EVs derived from untreated CRC cells. Sen-EVs, EVs derived from senescent CRC cells. **(C** and **D)** Western blot analysis of EVs markers and endoplasmic reticulum marker in whole cell lysates and EVs. **(E)** Concentration and size distribution of EVs assessed by NTA. **(F)** Quantitation of the relative EVs number per cell. The relative number of EVs per cell = (particle size concentration of EVs) × (volume of EVs)/total number of cells. **(G)** CRC cells were incubated with PKH67-labeled Sen-EVs for 18 h, and the uptaken of Sen-EVs was detected by fluorescence microscopy. Green fluorescence indicated PKH67, and blue fluorescence reflected nuclear staining with Hoechst 33342. Data represented the mean ± standard deviation of at least 3 independent experiments. *p < 0.05, **p < 0.01, and ***p < 0.001

Current research suggests that almost all types of cells have the capacity to release EVs, while it remains to be deciphered on the secretion of EVs by therapy-induced STCs. Therefore, we isolated EVs from the culture medium using differential ultracentrifugation (Fig. 4A). EVs derived from senescent CRC cells were defined as Sen-EVs, and EVs from untreated CRC cells as Ctrl-EVs. TEM revealed that the Sen-EVs and Ctrl-EVs displayed typical double-layered membrane vesicles (Fig. 4B). The positive markers of EVs were detected in whole cell lysate and EVs, while the endoplasmic reticulum marker Calnexin was undetectable in EVs (Fig. 4C and D). The particle sizes between Sen-EVs and Ctrl-EVs were similar (Fig. 4E and S2E). Intriguingly, the relative number of Sen-EVs was markedly higher than Ctrl-EVs (Fig. 4F and S2F). Sen-EVs labeled with PKH67 were co-cultured with CRC cells, and the internalization of Sen-EVs was observed after 18 h, which indicated that Sen-EVs were actively incorporated by cancer cells in vitro (Fig. 4G). Above all, these results indicated that therapy-induced STCs secreted a greater number of EVs, which could be internalized by recipient cancer cells.

### EVs secreted from STCs further enhanced the malignant biological behaviors of recipient cancer cells

**Fig. 5:**
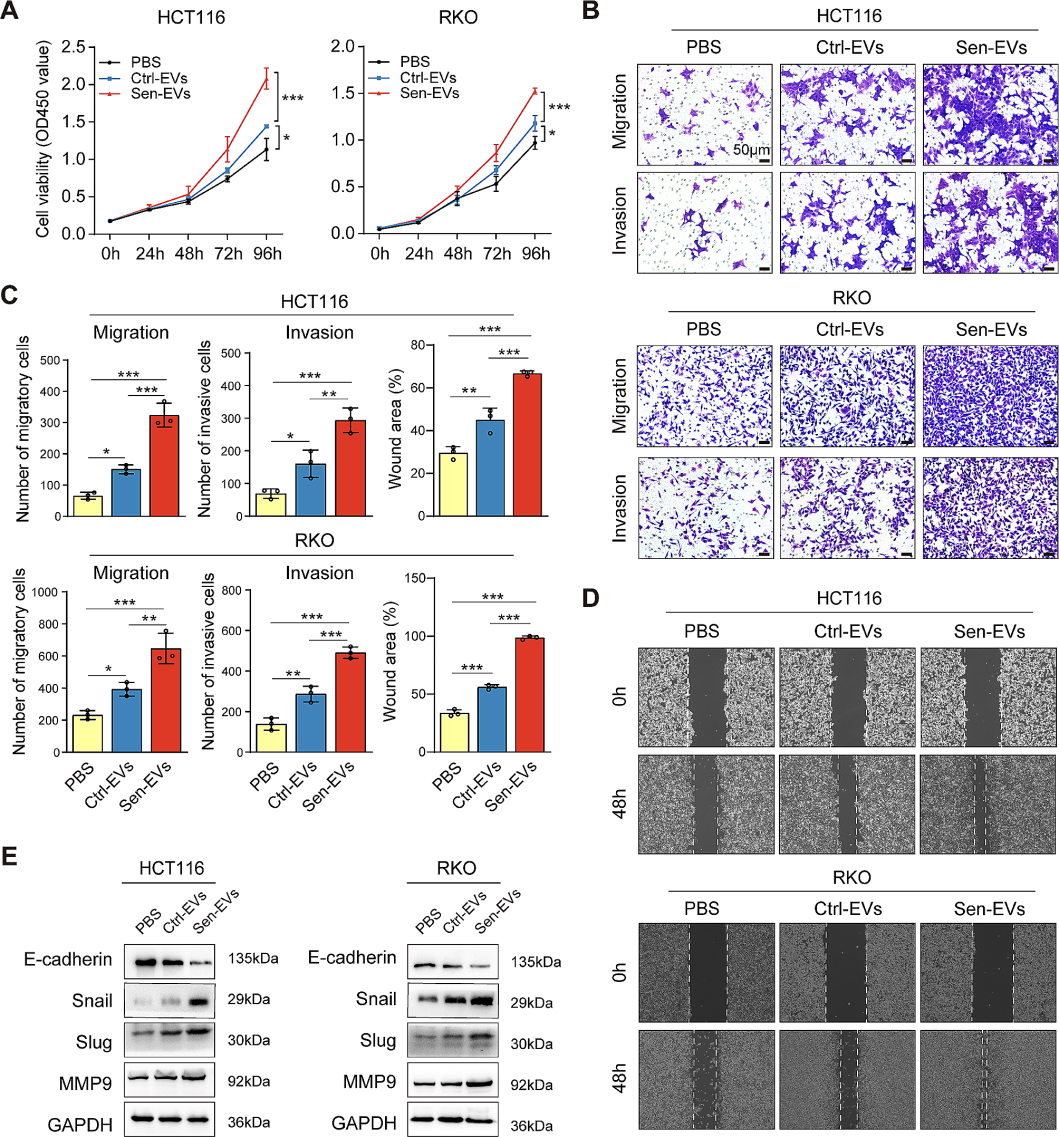
EVs secreted from STCs further enhanced the malignant biological behaviors of recipient cancer cells. **(A)** CRC cells were incubated with or without EVs (15 µg/ml) and then analyzed for cell viability via CCK-8 assay. **(B-E)** CRC cells were cultured with or without EVs (15 µg/ml) for 48 h. Ctrl-EVs, EVs derived from untreated CRC cells. Sen-EVs, EVs from senescent CRC cells. **(B)** Cell migration and invasion abilities were analyzed by transwell assay. **(C)** Quantification of migratory cells, invasive cells, and wound-healing rates. **(D)** Representative wound-healing images of CRC cells. **(E)** E-cadherin, Snail, Slug, and MMP9 protein levels. Data represented the mean ± standard deviation of at least 3 independent experiments. *p < 0.05, **p < 0.01, and ***p < 0.001

Noteworthy, recent evidence has shown the tumor-promoting effect of EVs in multiple cancers. Therefore, we investigated the potential influence of Sen-EVs on the CRC cells in vitro. Compared to PBS treatment, both Sen-EVs and Ctrl-EVs significantly promoted CRC cell proliferation, and Sen-EVs exhibited a stronger growth-promoting ability than Ctrl-EVs (Fig. 5A). Additionally, Sen-EVs and Ctrl-EVs enhanced the migration and invasion abilities of cancer cells, and more importantly Sen-EVs showed an even greater effect (Fig. 5B-D). Compared to CRC cells treated with PBS or Ctrl-EVs, the expression of E-cadherin in CRC cells incubated with Sen-EVs was decreased, and the expression of Snail, Slug, and MMP-9 was elevated, which further suggested the promotional effect of Sen-EVs on the migration and invasion of CRC cells (Fig. 5E). Collectively, our findings suggested that EVs released by STCs further enhanced the malignant phenotype of recipient CRC cells.

### SERPINE1 was enriched in EVs secreted from STCs

**Fig. 6:**
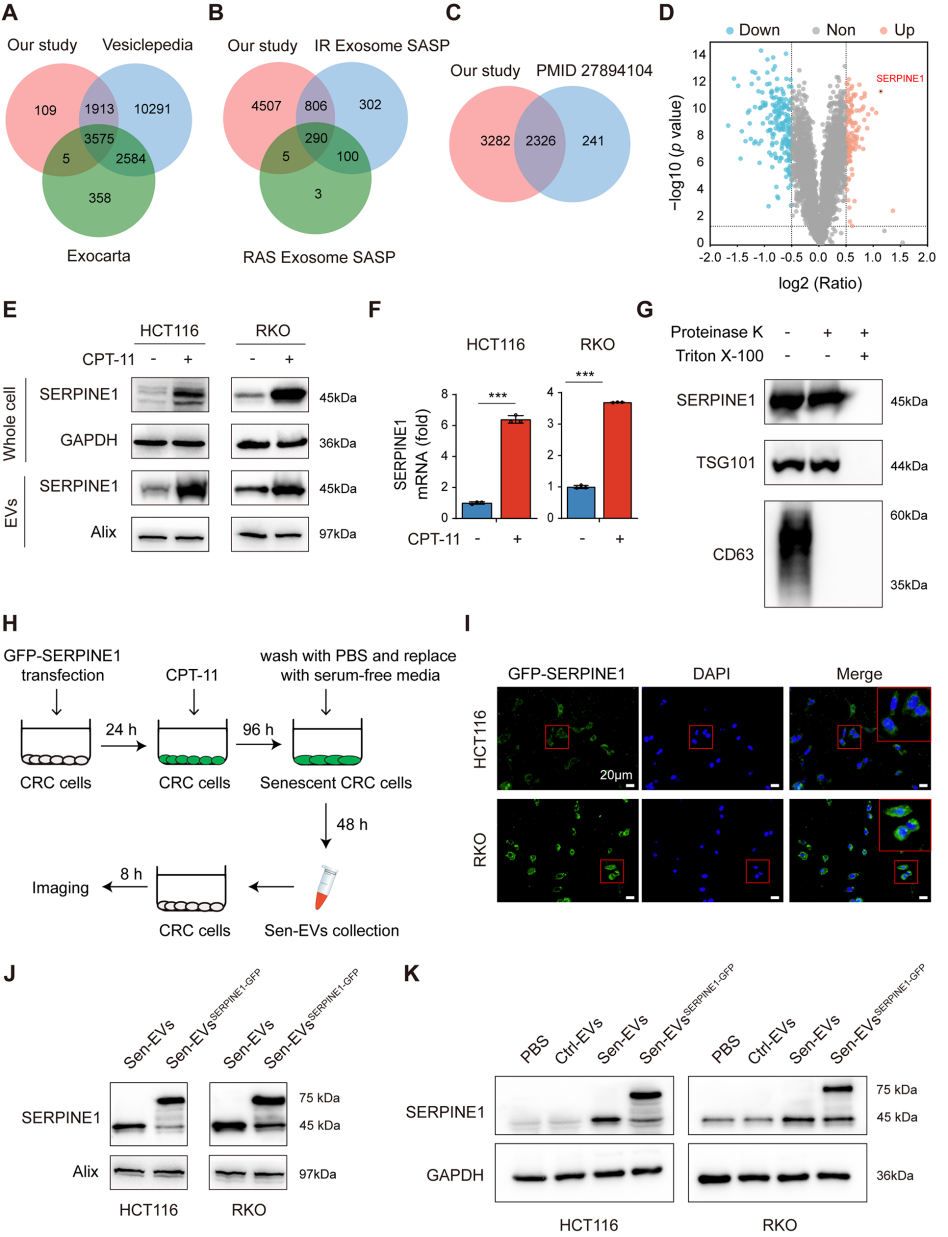
SERPINE1 was enriched in EVs secreted from STCs. **(A)** Venn diagram illustrating the similarities and differences between our study and ExoCarta and Vesiclepedia databases. **(B)** Venn diagram showing overlaps between our study and the SASPAtlas. **(C)** Venn diagram depicting the proteins detected in our study and those detected from the HCT116 cell line in the NCI-60. **(D)** Volcano plot of differentially expressed proteins between Ctrl-EVs and Sen-EVs. **(E** and **F)** CRC cells were treated with CPT-11 for 96 h to induce senescence, and untreated CRC cells were used as a control. **(E)** SERPINE1 protein level in whole cell lysates and EVs. **(F)** SERPINE1 mRNA level in CRC cells. **(G)** EVs derived from STCs were treated with proteinase K in the presence or absence of Triton X-100 and analyzed by western blot. **(H)** Scheme of cells cultured with Sen-EVs^SERPINE1−GFP^. CRC cells were plated in dishes and transfected with GFP-SERPINE1. After 24 h, cells were treated with CPT-11 for 96 h to induce senescence. The cultured cells were washed twice with PBS and cultured in serum-free DMEM for 48 h, then the culture media was collected for Sen-EVs^SERPINE1−GFP^ isolation. The recipient cells were co-incubated with the Sen-EVs^SERPINE1−GFP^ (15 µg/ml) for 8 h and photographed under a fluorescent microscope. **(I)** Fluorescence imaging of CRC cells treated with Sen-EVs^SERPINE1−GFP^. **(J)** SERPINE1 protein level in Sen-EVs and Sen-EVs^SERPINE1−GFP^. **(K)** CRC cells were incubated with EVs (15 µg/ml) or PBS for 48 h and the protein expression of SERPINE1 was detected. Data represented the mean ± standard deviation of at least 3 independent experiments. *p < 0.05, **p < 0.01, and ***p < 0.001

To assess the protein cargo inside EVs secreted from STCs, iTRAQ-labeling quantitative proteomic analysis was performed. We identified 5608 proteins, including the markers of EVs such as CD9, Alix, and TSG101 (Table S5). We compared the proteins detected in our proteomic analysis with the protein cargo in Vesiclepedia and ExoCarta databases [26, 27]. Over 90% of the EVs proteins in our experiment were common among those in the databases, providing a validation for the efficiency and accuracy of our isolation technique (Fig. 6A). Additionally, Basisty et al. presented a quantitative assessment of the SASPs (www.SASPAtlas.com) induced by various stimuli [28]. They analyzed both the EVs SASP (eSASP) and the soluble SASP (sSASP) of the senescent stromal cells [28]. The protein cargo identified in our proteomic analysis was compared with the eSASP in SASPAtlas. However, less than 20% of common proteins were identified, which may be explained by the senescence inducer- and cell type-dependent characteristics of SASP (Fig. 6B) [10]. Recently, a similar proteomic analysis was performed in 60 cancer cell lines, including HCT116. We found that more than 40% of the proteins detected in our study overlap with this study (Fig. 6C) [29].

To improve the confidence of protein quantification, we only analyzed proteins with more than three unique spectra. 119 proteins were increased, and 169 proteins were decreased in EVs secreted from senescent HCT116 cells compared to EVs from non-senescent cells (Fig. 6D). In particular, our attention was captured by serpin family E member 1 (SERPINE1), the second most enriched protein in Sen-EVs, which was also available in both Vesiclepedia and ExoCarta databases. SERPINE1, which belongs to the serine protease inhibitor (SERPIN) superfamily, is primarily known as an inhibitor of fibrinolysis via blocking the proteolytic activity of plasminogen activator. Moreover, a pivotal role of SERPINE1 in tumor development, such as metastasis, angiogenesis, and therapy-resistance, has also been found [30, 31]. To verify the proteomic analysis of EVs, we evaluated the expression of SERPINE1 in CRC cells and EVs and found that SERPINE1 was significantly increased in STCs and Sen-EVs (Fig. 6E, F and S2G). Proteinase K assay was employed to validate that SERPINE1 was indeed contained within EVs and to eliminate the possibility that SERPINE1 was an extracellular contaminant [32]. SERPINE1, similar to the luminal protein TSG101, was not sensitive to the digestion of proteinase K. In contrast, the transmembrane protein CD63 was digested by proteinase K. After Triton X-100 treatment, all proteins were sensitive to proteinase K digestion (Fig. 6G). Thus, we substantiated that SERPINE1 was a luminal cargo protein in Sen-EVs. To validate the SERPINE1 protein in Sen-EVs was transferred into recipient cancer cells, SERPINE1 tagged GFP (GFP-SERPINE1) was transfected into CRC cells, and senescence was induced by CPT-11 and EVs were isolated (defined as Sen-EVs^SERPINE1−GFP^). Then the recipient CRC cells were co-cultured with Sen-EVs^SERPINE1−GFP^ for 8 h (Fig. 6H). We found that GFP-SERPINE1 packaged by Sen-EVs were directly assimilated into the recipient cells (Fig. 6I). Additionally, western blot result showed the expression of SERPINE1-GFP (75 kDa) in the Sen-EVs^SERPINE1−GFP^ (Fig. 6J). Moreover, compared to CRC cells cultured with PBS or Ctrl-EVs, cells cultured with Sen-EVs and Sen-EVs^SERPINE1−GFP^ showed a significantly elevated expression of SERPINE1, which further implied SERPINE1 was enriched in Sen-EVs and could be internalized by recipient CRC cells (Fig. 6K). All together, these results suggested that SERPINE1 was enriched in EVs secreted from STCs.

### SERPINE1 was responsible for the tumor-promoting effect of EVs secreted by STCs

**Fig. 7:**
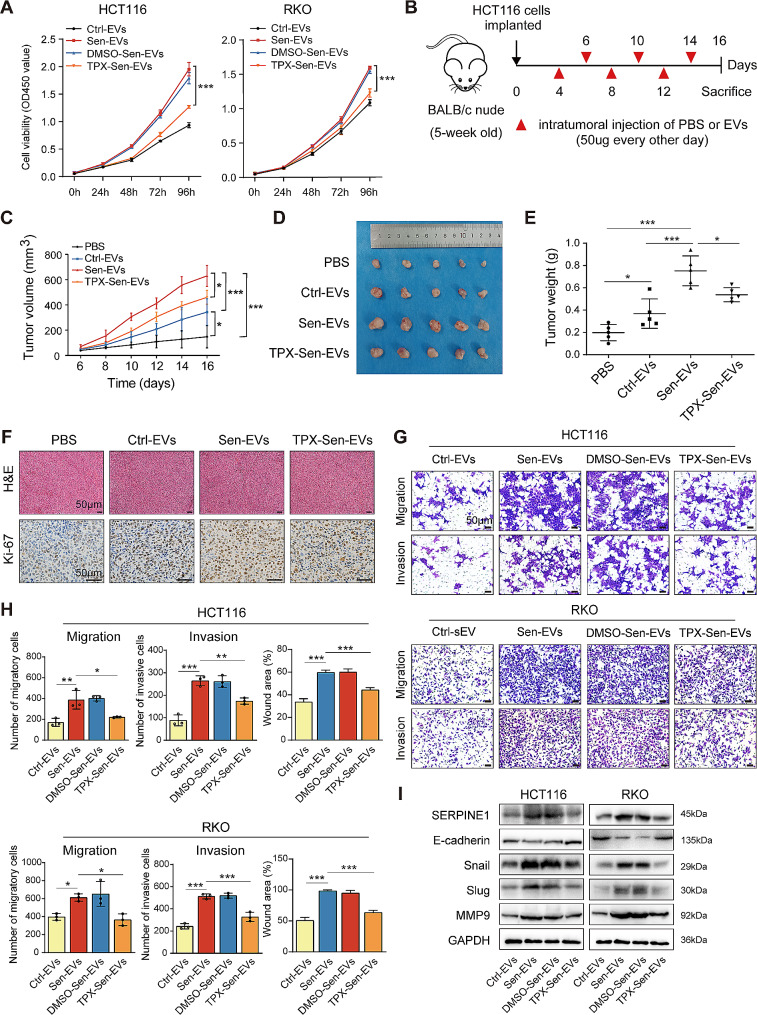
SERPINE1 was responsible for the tumor-promoting effect of EVs secreted from STCs. **(A)** Cell viability of CRC cells cultured with EVs at the indicated time points. **(B)** Schematic illustration of the establishment of the in vivo tumor model. **(C)** The tumor growth curve. **(D)** Images of the excised xenograft tumors. **(E)** Measurement of tumor weight. **(F)** H&E and IHC staining of Ki-67. **(G-I)** CRC cells were cultured with EVs (15 µg/ml) for 48 h. **(G)** The migration and invasion abilities of recipient CRC cells were analyzed by transwell assay. **(H)** Quantification of migratory cells, invasive cells, and wound-healing rates. **(I)** SERPINE1, E-cadherin, Snail, Slug, and MMP9 protein levels. Data represented the mean ± standard deviation of at least 3 independent experiments. *p < 0.05, **p < 0.01, and ***p < 0.001

Given the above findings, we asked whether SERPINE1 was responsible for the tumor-promoting effect of Sen-EVs. CRC cells were treated with TPX, a selective inhibitor of SERPINE1, to inhibit the expression of SERPINE1, and EVs were harvested (defined as TPX-Sen-EVs). The growth rate of CRC cells cultured with TPX-Sen-EVs was markedly reduced compared to those treated with Sen-EVs (Fig. 7A). We further conducted tumor xenograft experiments to confirm the growth-promoting effect of Sen-EVs in vivo (Fig. 7B). Both Sen-EVs and Ctrl-EVs significantly promoted tumor growth, while Sen-EVs exhibited a stronger growth-promoting ability than Ctrl-EVs (Fig. 7C-E). Meanwhile, compared to the Sen-EVs group, tumor growth and tumor weight of TPX-Sen-EVs-treated group were markedly decreased (Fig. 7C-E). The results of Ki-67 staining further validated the growth-promoting effect of Sen-EVs (Fig. 7F). Moreover, the results of the transwell and wound healing assays indicated that the promotional effects of Sen-EVs on cell migration and invasion were attenuated by SERPINE1 inhibition (Fig. 7G and H). Consistent with this, changes in the expression of epithelial-to-mesenchymal transition (EMT) markers indicated that TPX treatment impaired the EMT-promoting ability of Sen-EVs (Fig. 7I). Above all, our results revealed that SERPINE1 was in large part responsible for the tumor-promoting effect of Sen-EVs.

### SERPINE1 promoted NF-κB p65 nuclear translocation

**Fig. 8:**
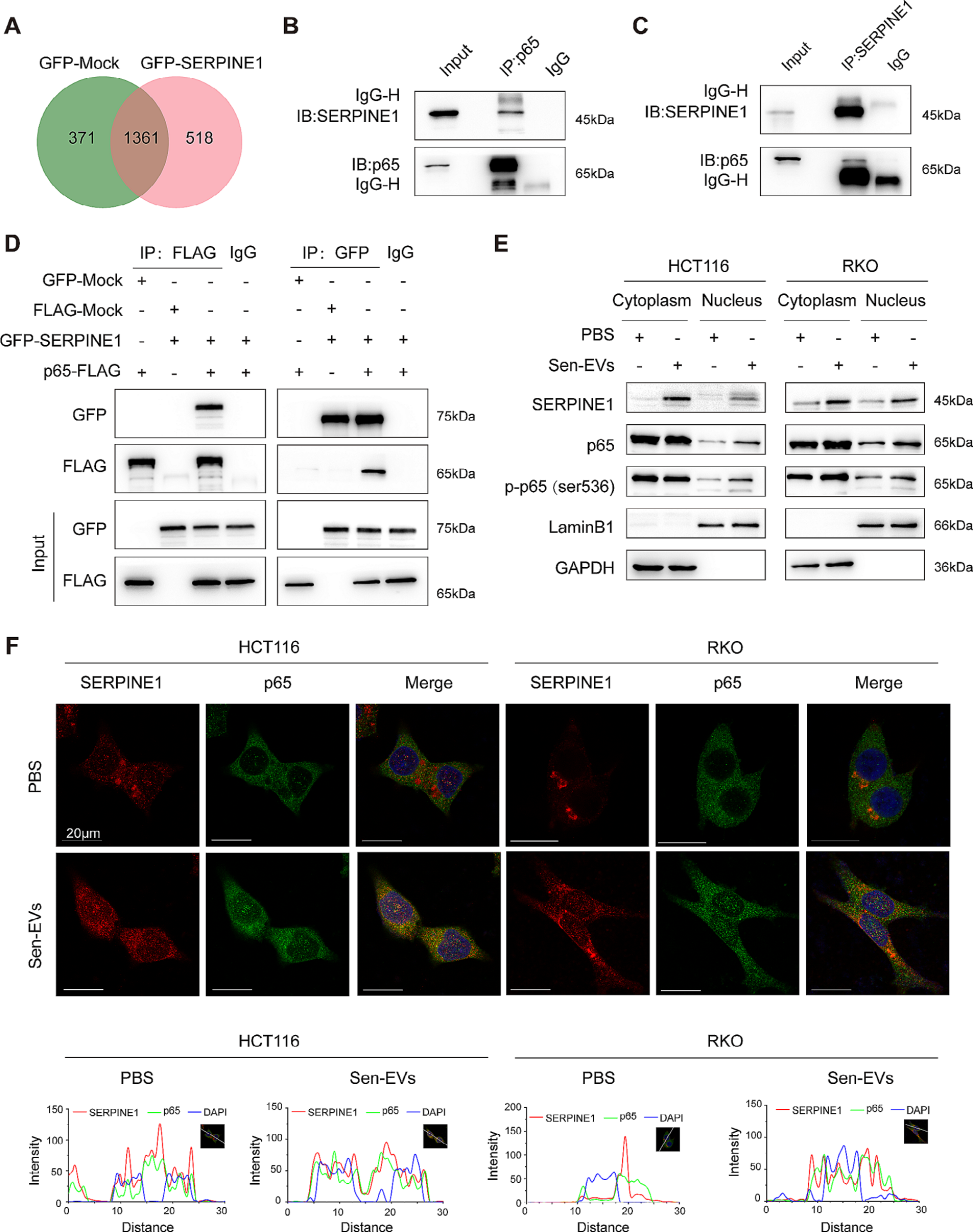
SERPINE1 promoted NF-κB p65 nuclear translocation. **(A)** GFP-SERPINE1 plasmids were transfected into 293T cells, the GFP-Mock plasmids were used as control. We immunoprecipitated extracts from whole cell lysates with anti-GFP antibody and analyzed the resulting complexes by LC-MS/MS. Venn diagram showed the proteins pulled down in GFP-SERPINE1 group and GFP-Mock group. **(B** and **C)** Co-IP of endogenous interaction of p65 and SERPINE1 in RKO cells. **(D)** Co-IP of exogenous interaction of p65 and SERPINE1 in 293T cells. **(E** and **F)** CRC cells were co-cultured with Sen-EVs (15 µg/ml) for 48 h. **(E)** Cellular distribution of SERPINE1, p65, and p-p65 was detected. **(F)** Representative images of immunofluorescence staining displaying the co-localization of SERPINE1 (red) and p65 (green). DAPI: blue. Line chart of fluorescence signal positioning analysis

It is well established that SERPINE1 has three protein-binding domains and interacts with LRP1, vitronectin, and PLAU/PLAT, respectively (Fig. S3A). Most research focused on the interplay of SERPINE1 with these three proteins in the extracellular space, while its intracellular function still needs to be explored. Co-IP assay and MS analysis were performed to identify the proteins that potentially bind to SERPINE1. 518 proteins exclusively pulled down in the GFP-SERPINE1 group in total (Fig. 8A and Table S6). Among these proteins, SERPINE1 had the highest abundance. Several proteins, such as LRP1 and PLAT, previously reported binding to SERPINE1 were detected (Fig. S3B and C). Interestingly, p65, a key subunit of NF-κB, was also detected in the GFP-SERPINE1 group (Fig. S3D). It is well known that the NF-κB signaling pathway regulates a diverse range of biological processes including inflammatory response, cellular growth, and cancer metastasis [33, 34]. Thus, we focused on the interaction between p65 and SERPINE1. The endogenous and exogenous interaction between SERPINE1 and p65 were verified by Co-IP assay in RKO cells and 293T cells, respectively (Fig. 8B-D).

Next, we explored whether SERPINE1 could promote the nuclear translocation of p65. CRC cells were co-cultured with Sen-EVs and cellular distribution of SERPINE1 and p65 was detected. As speculated, SERPINE1 was observed in the cytoplasm as well as the nucleus. Furthermore, the nuclear distribution of SERPINE1, p65 and p-p65 in CRC cells was increased after Sen-EVs stimulation (Fig. 8E). These results suggested that SERPINE1, which was released by Sen-EVs, transferred to the nucleus together with p65. We further performed immunofluorescence to validate the interaction between p65 and SERPINE1. The results exhibited an enhancement of p65 and SERPINE1 signal within the nucleus after stimulation with Sen-EVs, and the co-localization of SERPINE1 and p65 was also verified (Fig. 8F).

### SERPINE1 expression was elevated in tumors after anti-cancer treatment and predicted poor prognosis

**Fig. 9:**
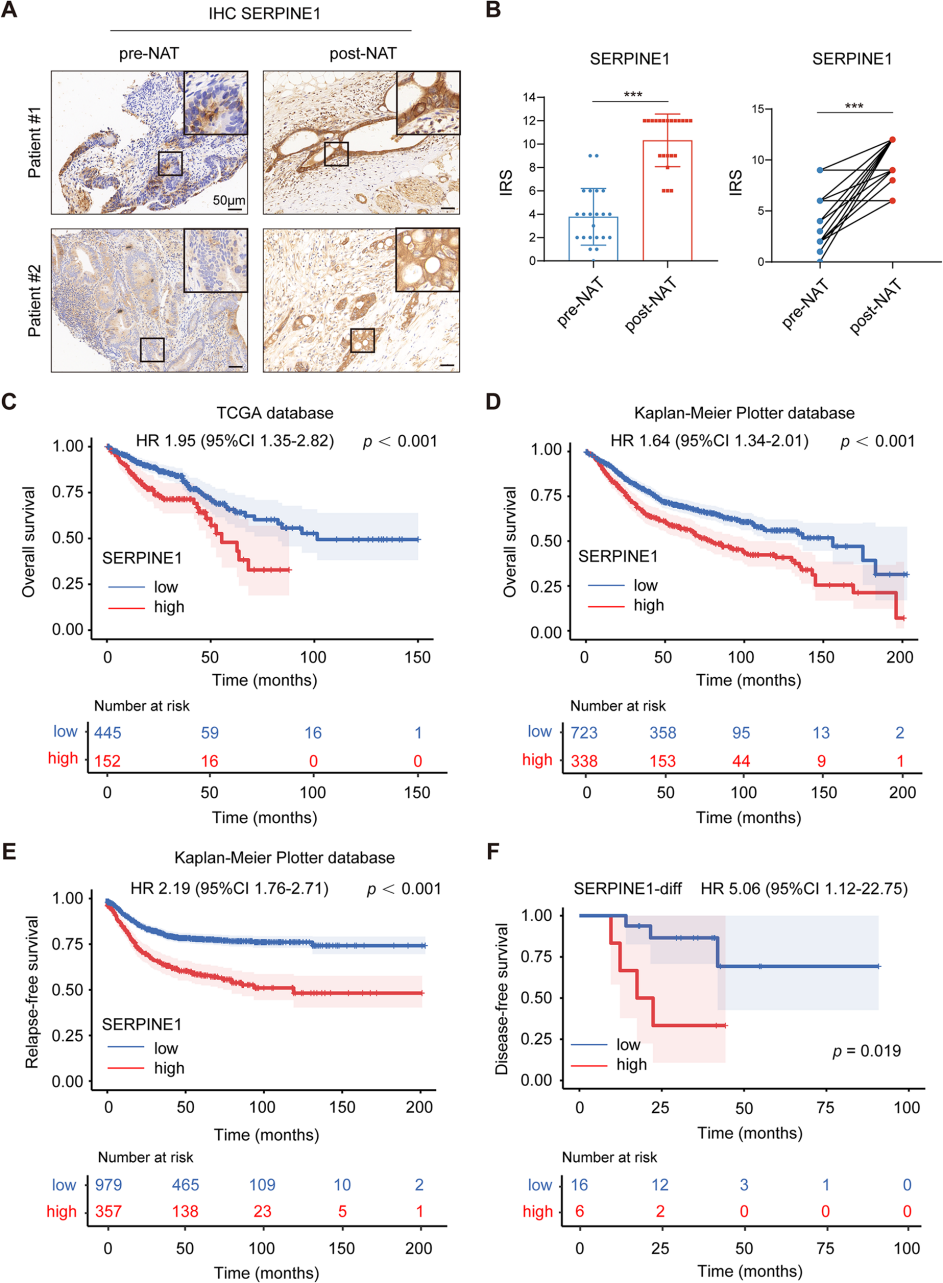
SERPINE1 expression was elevated in tumors after NAT and predicted poor prognosis in cohort 1. **(A)** IHC staining of SERPINE1 in matched LARC tissues. **(B)** The IRS of SERPINE1 in matched LARC tissues. **(C)** OS of CRC patients from the TCGA database. **(D)** OS and **(E)** RFS of colon patients from the Kaplan-Meier plotter database. **(F)** DFS of 22 LARC patients in cohort 1. The plot was generated according to the difference between pre-NAT IRS and post-NAT IRS of SERPINE1 (SERPINE1-diff). *p < 0.05, **p < 0.01, and ***p < 0.001

To evaluate the clinical significance of SERPINE1 in CRC, tumor tissues from cohort 1 and cohort 2 were analyzed for the expression of SERPINE1 using IHC staining. SERPINE1 expression significantly increased after anti-cancer treatment (Fig. 9A, B and S4A, B). Furthermore, analysis of the TCGA database and Kaplan-Meier plotter database indicated that patients with higher expression of SERPINE1 displayed worse overall survival (OS) (Fig. 9C and D) [35]. Additionally, the Kaplan-Meier curve also revealed a significant correlation of high SERPINE1 expression with shorter relapse-free survival (RFS) (Fig. 9E). We analyzed the difference between pre-NAT IRS and post-NAT IRS of SERPINE1 (SERPINE1-diff) and found that patients with a greater change of SERPINE1 expression in LARC tissues after NAT correlated with reduced DFS (Fig. 9F). Similarly, a negative relationship between the SERPINE1-diff and PFS was observed in mCRC patients of cohort 2 (Fig. S4C). Altogether, these results highlighted the clinical prognostic significance of SERPINE1 in CRC.

## Discussion

The induction of senescence in cancer cells has been recognized as a common outcome of conventional anti-cancer treatments, such as chemotherapy and radiotherapy [4]. Senescence is originally considered as a tumor-suppressive mechanism through halting cancer cell proliferation and promoting anti-tumoral immune surveillance. Emerging evidence, however, suggests that the SASP of persistent STCs can promote the malignant behaviors of neighboring cancer cells via multiple mechanisms, including (i) secretion of MMPs that degrade the extracellular matrix contributing to the proliferation and invasion of cancer cells, (ii) increased tumor angiogenesis via the secretion of vascular endothelial growth factor (VEGF), and (iii) establishment of an immunosuppressive tumor microenvironment through the recruitment of immune suppressor cells [11, 36]. Apparently, the SASP factors are predominant mediators of the tumor-promoting functions of STCs. Of note, EVs are recently emerged as a novel component of the SASP and mediate the pro-tumorigenic effects of the SASP [10]. To the best of our knowledge, most of the current literature focuses on the EVs derived from stromal cells, such as mesenchymal stromal cells and cancer-associated fibroblasts [18, 19, 37]. Nevertheless, the effect of senescent cancer cell-derived EVs on tumor progression still remains to be deciphered. We thus investigated whether TIS in CRC cells might drive tumor progression via the development of EVs.

We found that both chemotherapeutic agent CPT-11 and IR could trigger senescence in CRC cells. More importantly, chemotherapy and radiotherapy potently induced cancer cell senescence in vivo, as evidenced by the up-regulated expression of p16 and p21 in tumor tissues from CRC patients who received anti-cancer treatment. Furthermore, we found a small fraction of cancer cells stained positive for p16 and p21 in tumor tissues prior to anti-cancer treatment, suggesting that CRC cells in vivo could undergo senescence spontaneously, as previously reported in lymphoma and breast cancer [38, 39]. In addition, our present study revealed occurrence of senescence mainly in the cancer cells, not the stromal cells, as a previous study described [40]. By contrast, a recent study observed extensive cellular senescence occurrence in the stromal cells of prostate tumor, rather than cancer cells [19]. This different distribution of senescent cells may be attributed to the difference in tumor type and treatment method. Most importantly, we evaluated the relationship between TIS and the clinical prognosis of CRC patients. We found that the greater up-regulation of p16 and p21 expression after anti-cancer treatment correlated with reduced DFS and PFS. However, the number of patients included in our study was limited. Additionally, because of the short follow-up period, we for the being time could not assess the influence of TIS on OS. More patients and longer follow-up time are needed to further validate our findings in the future.

SASP, the most distinctive feature of senescent cells, exerts tumor-promoting effects on surrounding cancer cells in a non-cell-autonomous fashion. Indeed, we found that senescent CRC cells induced by CPT-11 had a robust ability of the secretion of the SASP. Interestingly, in addition to soluble secretory proteins, senescent cells (in particular stromal cells in tumors) have been proven to release EVs promoting tumor progression. Moreover, recent studies have shown that cancer cells are also able to secrete EVs, which contribute to tumor development. However, it remains unknown on the potential for the production of EVs by senescent CRC cells. In this study, we for the first time confirmed that senescent CRC cells induced by CPT-11 and irradiation not only could release EVs but also exhibited significantly increased secretion of EVs compared to untreated CRC cells. Furthermore, EVs derived from STCs displayed a potent tumor-promoting activity both in vitro and in vivo. Our results offer further evidence of the critical role of cancer cell senescence in tumor promotion and strengthen the understanding of EVs as a new player to mediate the pro-tumorigenic function of the SASP.

We employed iTRAQ-labeling quantitative proteomic analysis to explore the EVs cargo released by STCs and found that SERPINE1 was significantly enriched in STCs-derived EVs. Targeting SERPINE1 with PTX attenuated the tumor-promoting function of STCs-derived EVs. SERPINE1 is an important SASP factor [41, 42]. In recent years, SERPINE1 has been observed to be enriched in EVs from malignant ascites, glioblastoma cells, and brain endothelial cells [43–45]. Here we reported for the first time that SERPINE1 was transferred from STCs to non-STCs via EVs, thereby contributing to tumor progression. It should be noted that since SASP expression in cancer is heterogeneous and influenced by cell origin, whether SERPINE1 is also enriched and functional in EVs from other types of cancer cells warrants further investigation [46].

Previous research demonstrated that SERPINE1 predominantly localizes in the extracellular space and contributes to tumor proliferation and metastasis [47, 48]. However, it was not until recently that the role of intracellular SERPINE1, especially nuclear SERPINE1, was reported [49, 50]. In bladder cancer cells, SERPINE1 was observed to be localized in the nucleus and act as a transcriptional co-regulator [49]. The nuclear distribution of SERPINE1 in breast cancer cells was elevated after IR exposure to promote DNA double-strand breaks repair [50]. However, how nuclear entry is achieved for SERPINE1 remains unknown. The best-characterized nuclear import pathways are mediated by importin-cargo interactions in which importins interact with nuclear localization signal (NLS)-containing cargos [51]. But according to the PSORT II prediction software package (https://psort.hgc.jp/form2.html), SERPINE1 contains no currently identifiable NLS [52]. To identify the potential binding partners of SERPINE1, LC-MS/MS analysis was performed. p65, along with other known partners of SERPINE1, was detected. Further investigation suggested that SERPINE1 bound to p65 and promoted p65 nuclear translocation. Thus, we speculate that SERPINE1 may piggyback on NLS-containing proteins, such as p65, to gain nuclear entry. Alternatively, there exists some other nuclear import systems that is independent of importins, such as the RanGDP/AR complex-mediated nuclear import system [53]. Whether SERPINE1 could use the RanGDP/AR pathway or other pathways to enter the nucleus need further investigation. Notably, the underlying mechanism by which SERPINE1 promotes p65 nuclear translocation remains to be deciphered.

Notably, there are some limitations in our study. On one hand, we analyzed the occurrence of cellular senescence in vivo just through the combined evaluation of p16 and p21 expression without the detection of SA-β-Gal activity, which is the ‘gold standard’ of senescence marker. This is due to formalin-fixed paraffin-embedded tissues used in our study, which are not suitable for SA-β-Gal staining. On the other hand, we isolated EVs through differential ultracentrifugation, which is considered as the canonical method of EVs isolation. However, the yields of EVs separated by ultracentrifugation is low and the purity is intermediate [54]. Several isolation methods with higher yields and purity are needed in the future study.

## Conclusion

Overall, we provide in vivo evidence of the clinical prognostic implications of TIS. Our findings demonstrate that STCs, which abundantly exist in tumor tissues following chemotherapy and radiotherapy, are responsible for tumor progression by producing large amounts of EVs enriched in SERPINE1. Mechanistically, SERPINE1 binds to NF-κB p65, promotes its nuclear translocation, and activates the NF-κB signaling pathway. Our results reveal the tumor-promoting effect of EVs derived from STCs and pinpoint SERPINE1 as a therapeutic target to overcome TIS-associated progression in CRC.

### Electronic supplementary material

Below is the link to the electronic supplementary material.

Additional file 1: Table S1. The primers used in this study.

Additional file 2: Table S2. The primary antibodies used in this study.

Additional file 3: Table S3. The clinicopathologic features of LARC patients in cohort 1.

Additional file 4: Table S4. The clinicopathologic features of mCRC patients in cohort 2.

Additional file 5: Table S5. The proteins in EVs identified with proteomic analysis.

Additional file 6: Table S6. The SERPINE1 interacting partners identified with LC-MS/MS.

Additional file 7: Figure S1. CPT-11 inhibited cell growth and failed to activate apoptosis in CRC cells.

Additional file 8: Figure S2. IR induced senescence in HCT116 cells and increased the secretion of EVs enriched in SERPINE1.

Additional file 9: Figure S3. The string analysis and LC-MS/MS spectrum proteins potentially interact with SERPINE1.

Additional file 10: Figure S4. SERPINE1 expression was elevated in tumors after anti-cancer treatment and indicated poor prognosis (cohort 2).

Additional file 11: Supplementary Figure legends.

## Data Availability

The iTRAQ-based proteomics data and mass spectrometry data have been deposited to the ProteomeXchange Consortium (http://www.proteomexchange.org/) via the PRIDE partner repository with the dataset identifier PXD047062 and PXD047066, respectively.

## Abbreviations

CPT-11: Irinotecan
CRC: Colorectal cancer
EVs: Extracellular vesicles
SA-β-Gal: Senescence-associated beta-galactosidase
SASP: Senescence-associated secretory phenotype
SERPINE1: Serpin family E member 1
STCs: Senescent tumor cells
TPX: Tiplaxtinin
TIS: Therapy-induced senescence
