# Supplementary figures and images for: Therapy-induced senescent tumor cell-derived extracellular vesicles promote colorectal cancer progression through SERPINE1-mediated NF-κB p65 nuclear translocation
Source: Mol Cancer. 2024 Apr 4;23:70. doi: 10.1186/s12943-024-01985-1 (PMC10993572; doi:10.1186/s12943-024-01985-1)

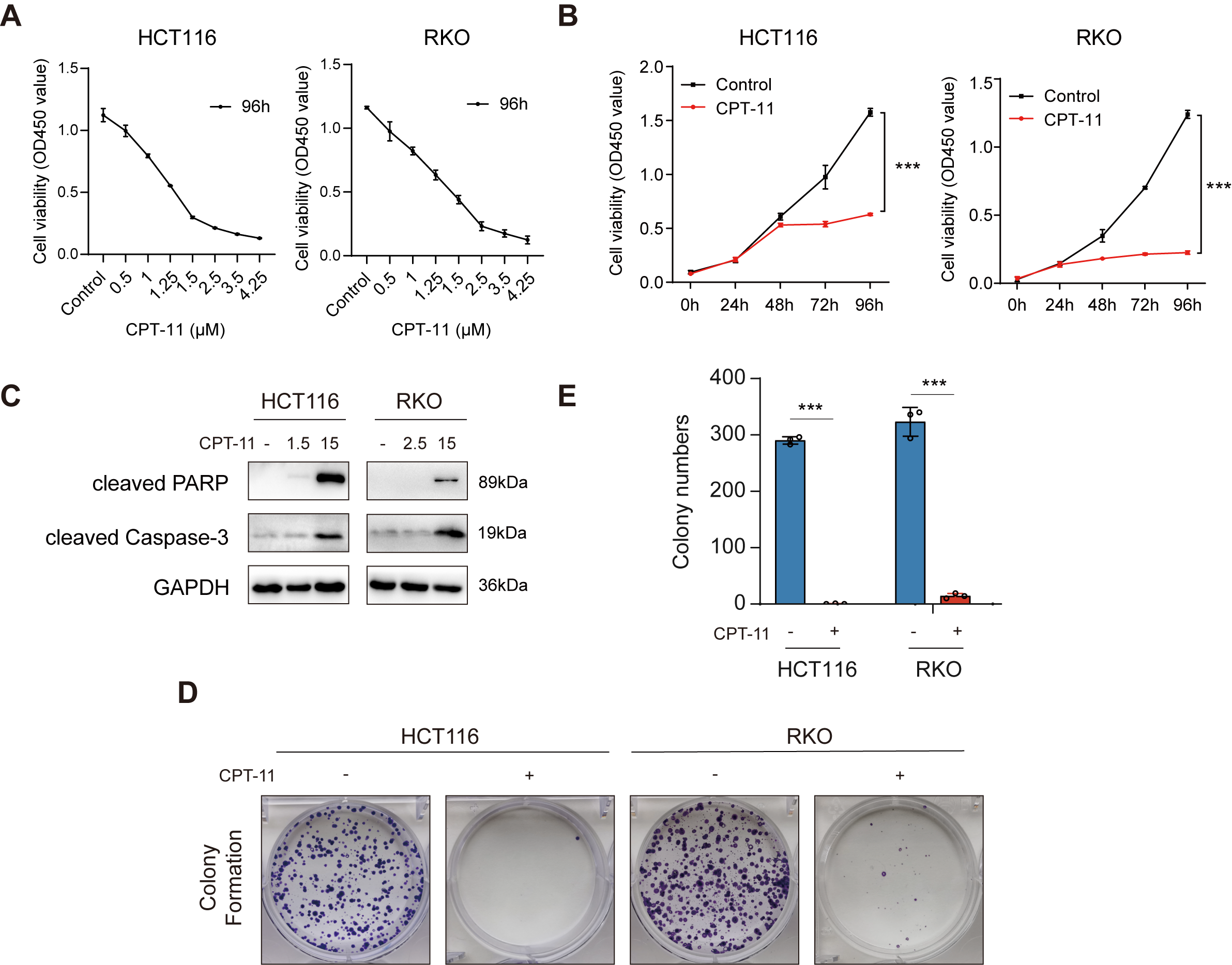

Supplement: Supplementary file 7 — Additional file 7: Figure S1. CPT-11 inhibited cell growth and failed to activate apoptosis in CRC cells. [file 12943_2024_1985_MOESM7_ESM.tif]

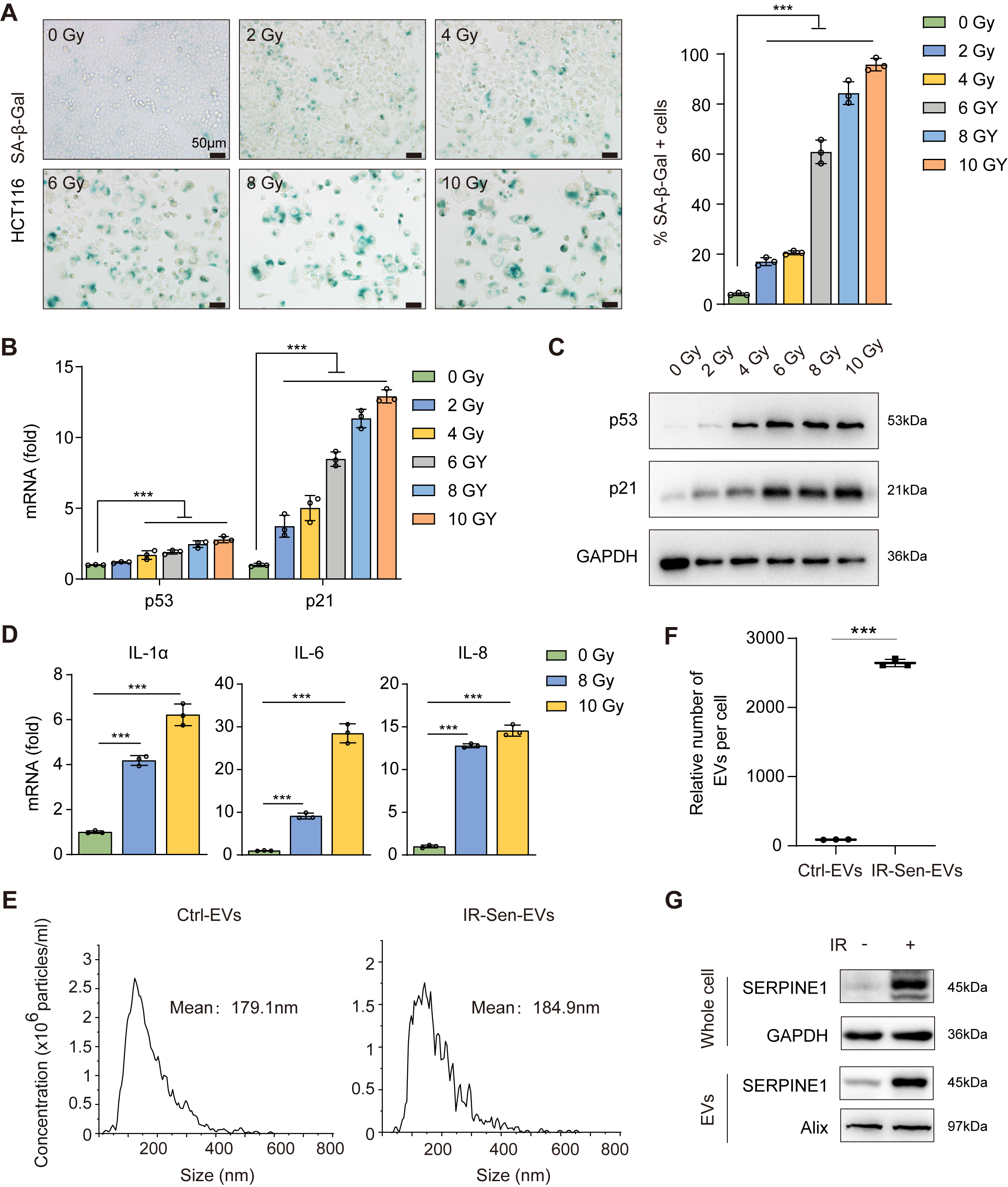

Supplement: Supplementary file 8 — Additional file 8: Figure S2. IR induced senescence in HCT116 cells and increased the secretion of EVs enriched in SERPINE1. [file 12943_2024_1985_MOESM8_ESM.tif]

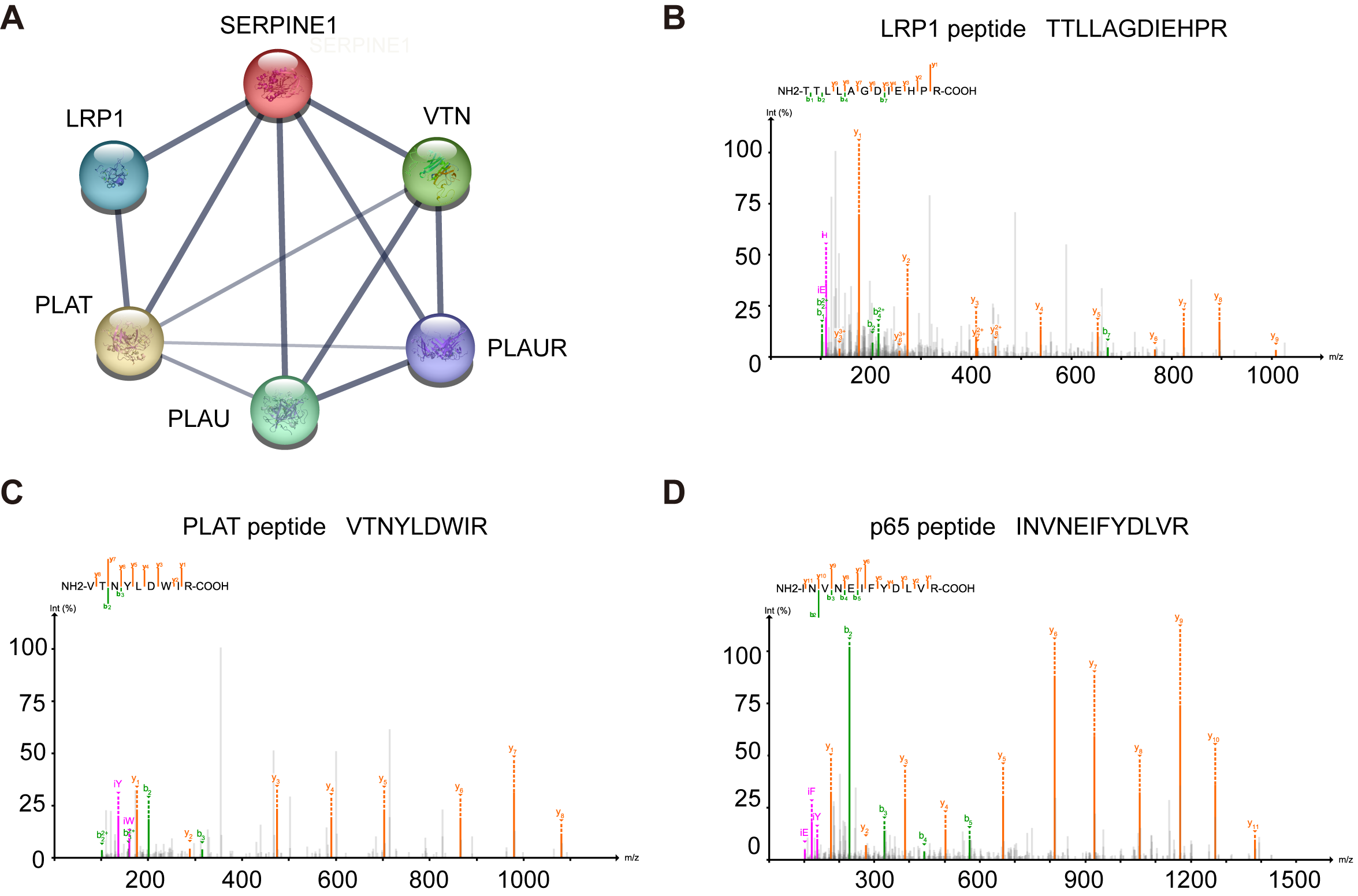

Supplement: Supplementary file 9 — Additional file 9: Figure S3. The string analysis and LC-MS/MS spectrum proteins potentially interact with SERPINE1. [file 12943_2024_1985_MOESM9_ESM.tif]

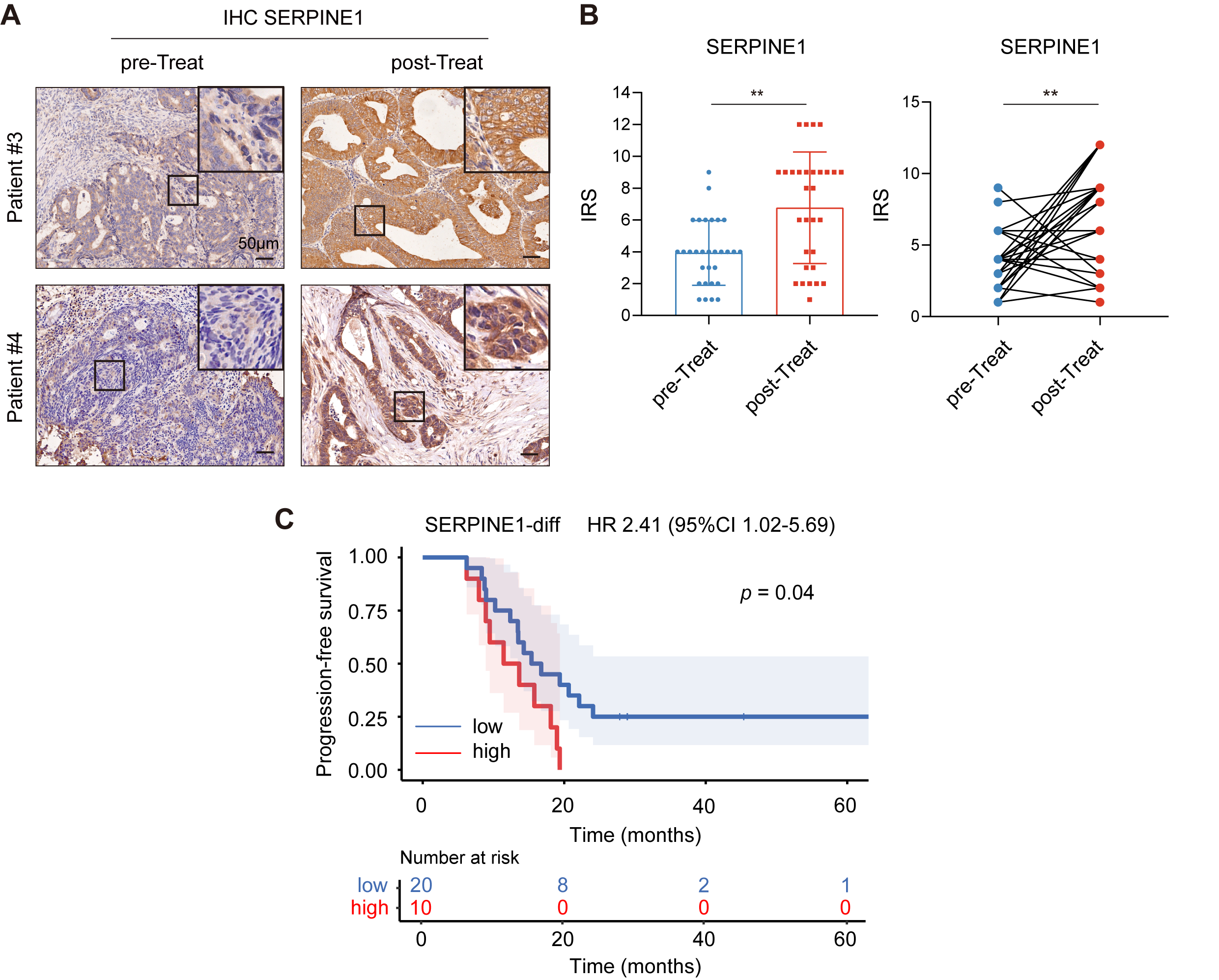

Supplement: Supplementary file 10 — Additional file 10: Figure S4. SERPINE1 expression was elevated in tumors after anti-cancer treatment and indicated poor prognosis (cohort 2). [file 12943_2024_1985_MOESM10_ESM.tif]
