# Supplementary material for: Therapy-induced senescent tumor cell-derived extracellular vesicles promote colorectal cancer progression through SERPINE1-mediated NF-κB p65 nuclear translocation
Source: Mol Cancer. 2024 Apr 4;23:70. doi: 10.1186/s12943-024-01985-1 (PMC10993572; doi:10.1186/s12943-024-01985-1)
Supplement: Supplementary file 11 — Additional file 11: Supplementary Figure legends. [file 12943_2024_1985_MOESM11_ESM.docx]

**Supplementary Figure legends**

**Supplementary Fig. S1. CPT-11 inhibited cell growth and failed to activate apoptosis in CRC cells. (A)** CRC cells treated with various concentrations of CPT-11 for 96 h were examined for cell viability using CCK-8 assay. **(B)** Cell viability of CRC cells treated with or without CPT-11 at the indicated time points was analyzed. **(C)** CRC cells treated with or without CPT-11 for 96 h were re-plated into 6-well plates and cultured for two weeks to perform colony formation assay. **(D)** Quantification of colony numbers. **(E)** CRC cells treated with or without CPT-11 at the indicated concentrations for 96 h were examined for the protein expression of cleaved PARP, cleaved Caspase-3, and a loading control GAPDH by western blot. Cells treated with 15 μM CPT-11 for 24 h were set as positive control. Data represented the mean ± standard deviation of at least 3 independent experiments. ******p* < 0.05, *******p* < 0.01, and ********p* < 0.001.

**Supplementary Fig. S2. IR induced senescence in HCT116 cells and increased the secretion of EVs enriched in SERPINE1. (A-D)** HCT116 cells were irradiated with the indicated doses and the induction of cellular senescence was detected. **(A)** Left panel: representative photographs of SA-β-Gal staining on IR-treated HCT116 cells. Right panel: quantification of SA-β-Gal-positive cells. **(B)** RT-qPCR analysis of the mRNA level of p53 and p21. **(C)** Immunoblotting blot analysis of the protein levels of p53 and p21. **(D)** mRNA levels of SASP factor. **(E-G)** HCT116 cells were exposed to 10 Gy irradiation and cultured for 96 h to allow the development of the senescent phenotype. Untreated HCT116 cells were cultured for 48 h and served as control. Ctrl-EVs, EVs secreted from HCT116 cells without IR treatment. IR-Sen-EVs, EVs isolated from senescent HCT116 cells induced by IR. **(E)** Concentration and size distribution of EVs assessed by NTA. **(F)** Quantitative comparison of the relative number of EVs secreted from HCT116 cells detected by NTA. **(G)** The protein expression of SERPINE1 in whole cell lysates and EVs. Data represented the mean ± standard deviation of at least 3 independent experiments. ******p* < 0.05, *******p* < 0.01, and ********p* < 0.001.

**Supplementary Fig. S3. The string analysis and LC-MS/MS spectrum proteins potentially interact with SERPINE1. (A)** String analysis of proteins found to interact with SERPINE1. **(B-D)** LC-MS/MS spectrum showing the peptides of LRP1, PLAT, and p65 pulled down by SERPINE1.

**Supplementary Fig. S4. SERPINE1 expression was elevated in mCRC tissues after irinotecan-based chemotherapy and predicted poor prognosis (cohort 2). (A)** IHC staining of SERPINE1 in matched mCRC tissues. pre-Treat, colonoscopy biopsies or surgically resected tumors before anti-cancer treatment. post-Treat, surgically resected tumors after irinotecan-based chemotherapy. **(B)** The IRS of SERPINE1. **(C)** Kaplan-Meier plots of PFS. The plot was generated according to the difference between pre-Treat IRS and post-Treat IRS of SERPINE1 (SERPINE1-diff). ******p* < 0.05, *******p* < 0.01, and ********p* < 0.001.
